# Mitofusin-2 boosts innate immunity through the maintenance of aerobic glycolysis and activation of xenophagy in mice

**DOI:** 10.1038/s42003-021-02073-6

**Published:** 2021-05-10

**Authors:** Prashanta Silwal, Jin Kyung Kim, Sang Min Jeon, June-Young Lee, Young Jae Kim, Yi Sak Kim, Yeji Seo, Jihye Kim, Soo Yeon Kim, Min Joung Lee, Jun Young Heo, Mi-Ja Jung, Hyun Sik Kim, Dong-Wook Hyun, Jeong Eun Han, Jake Whang, Yang Hoon Huh, Sang-Hee Lee, Won Do Heo, Jin-Man Kim, Jin-Woo Bae, Eun-Kyeong Jo

**Affiliations:** 1 Department of Microbiology, Chungnam National University School of Medicine, Daejeon, Korea; 2 Infection Control Convergence Research Center, Chungnam National University School of Medicine, Daejeon, Korea; 3 Department of Medical Science, Chungnam National University School of Medicine, Daejeon, Korea; 4 Department of Life and Nanopharmaceutical Sciences and Department of Biology, Kyung Hee University, Seoul, Korea; 5 Department of Medicine, University of California, San Diego, CA USA; 6 Department of Biological Sciences, Korea Advanced Institute of Science and Technology (KAIST), Daejeon, Korea; 7 Future Medicine Division, Korea Institute of Oriental Medicine, Daejeon, Korea; 8 Department of Biochemistry, Chungnam National University School of Medicine, Daejeon, Korea; 9 Korea Mycobacterium Resource Center (KMRC) & Basic Research Section, The Korean Institute of Tuberculosis (KIT), Cheongju, Korea; 10 Center for Research Equipment, Korea Basic Science Institute, Cheongju, Korea; 11 Department of Pathology, Chungnam National University School of Medicine, Daejeon, Korea

**Keywords:** Infection, Bacterial infection

## Abstract

Mitochondrial function and innate immunity are intimately linked; however, the mechanisms how mitochondrion-shaping proteins regulate innate host defense remains largely unknown. Herein we show that mitofusin-2 (MFN2), a mitochondrial fusion protein, promotes innate host defense through the maintenance of aerobic glycolysis and xenophagy via hypoxia-inducible factor (HIF)-1α during intracellular bacterial infection. Myeloid-specific MFN2 deficiency in mice impaired the antimicrobial and inflammatory responses against mycobacterial and listerial infection. Mechanistically, MFN2 was required for the enhancement of inflammatory signaling through optimal induction of aerobic glycolysis via HIF-1α, which is activated by mitochondrial respiratory chain complex I and reactive oxygen species, in macrophages. MFN2 did not impact mitophagy during infection; however, it promoted xenophagy activation through HIF-1α. In addition, MFN2 interacted with the late endosomal protein Rab7, to facilitate xenophagy during mycobacterial infection. Our findings reveal the mechanistic regulations by which MFN2 tailors the innate host defense through coordinated control of immunometabolism and xenophagy via HIF-1α during bacterial infection.

## Abstract (web summary)

Silwal, Kim et al. show that mitofusin-2 (MFN2), a mitochondrial fusion protein, promotes innate host defense through coordinated orchestration of immunometabolism and xenophagy via HIF-1α in macrophages during bacterial infection. This study provides insights into how mitochondria-shaping protein is involved in host defense against intracellular bacterial infection.

## Introduction

Mitochondria are recognized as crucial hubs for the orchestration of a variety of physiological functions including cell survival, growth, death, and metabolic homeostasis^1^. Morphological changes, i.e., mitochondrial fission and fusion, are critical for maintaining mitochondrial quality control through the removal of damaged mitochondria^2^. The dynamic mitochondrial morphological changes are tightly controlled by dynamin-related GTPases, which constitute the core system of mitochondrial fusion and fission cycles^3^. The mitofusin proteins 1 (MFN1), 2 (MFN2), and optic atrophy (Opa1) are involved in the processes of mitochondrial fusion, whereas dynamin-related protein 1 (Drp1) and mitochondrial fission 1 (Fis1) proteins participate in the mitochondrial fission processes^3^. An imbalance in mitochondrial fusion and fission is associated with various pathological diseases like type 2 diabetes^4^, Alzheimer’s disease^5^, and heart failure^6^. However, the protein functions involved in shaping mitochondrial morphology and how they are involved in modulating antimicrobial innate defenses remain largely unknown.

Among mitochondrion-shaping proteins, MFN2 is a major mitochondrial fusion protein that coordinates mitochondrial quality control^7,8^. MFN2 is required for controlling insulin signaling by modulating reactive oxygen species (ROS) and endoplasmic reticulum stress^9,10^. MFN2 is also involved in cytoprotection, as it interacts with the NAD-dependent deacetylating enzyme sirtuin 1 (SIRT1), which deacetylates MFN2^8^. Moreover, the association between MFN2 and NLRP3 is required for inflammasome activation in response to RNA viruses^11^. Recently, MFN2 functions in macrophages have been reported in terms of inflammatory responses during bacterial infection and sepsis^12^; however, the mechanisms by which MFN2 regulates innate host defense against intracellular bacterial infection remain to be characterized. In addition, whether MFN2 is involved in the regulation of immunometabolism and xenophagy, both of which are critically related to innate defense^13^, remains largely overlooked. Because macrophages are critical to the functioning of the innate immune response, we investigated the mechanistic role of MFN2 in macrophages using *Mfn2*^*f/f*^;*LysM Cre*^+^ (*Mfn2* CKO) mice and their littermate controls for innate host defense against mycobacterial and listerial infections. Here we report that MFN2 promoted macrophage inflammatory signaling through optimal induction of aerobic glycolysis via hypoxia-inducible factor (HIF)-1α, which is activated by mitochondrial respiratory chain complex I and ROS, triggered by bacterial infection. In addition, MFN2 was required for the activation of xenophagy against *Mycobacterium tuberculosis* (Mtb) infection through HIF-1α. Furthermore, MFN2 interaction with the late-endosomal protein Rab7 contributed to activation of xenophagy during mycobacterial infection. Overall, our findings demonstrate that MFN2 is a key coordinator of innate immune responses against intracellular bacterial infection through the maintenance of aerobic glycolysis and activation of xenophagy via HIF-1α.

## Results

### MFN2 is required for host antimicrobial responses during mycobacterial and listerial infection

**Fig. 1:**
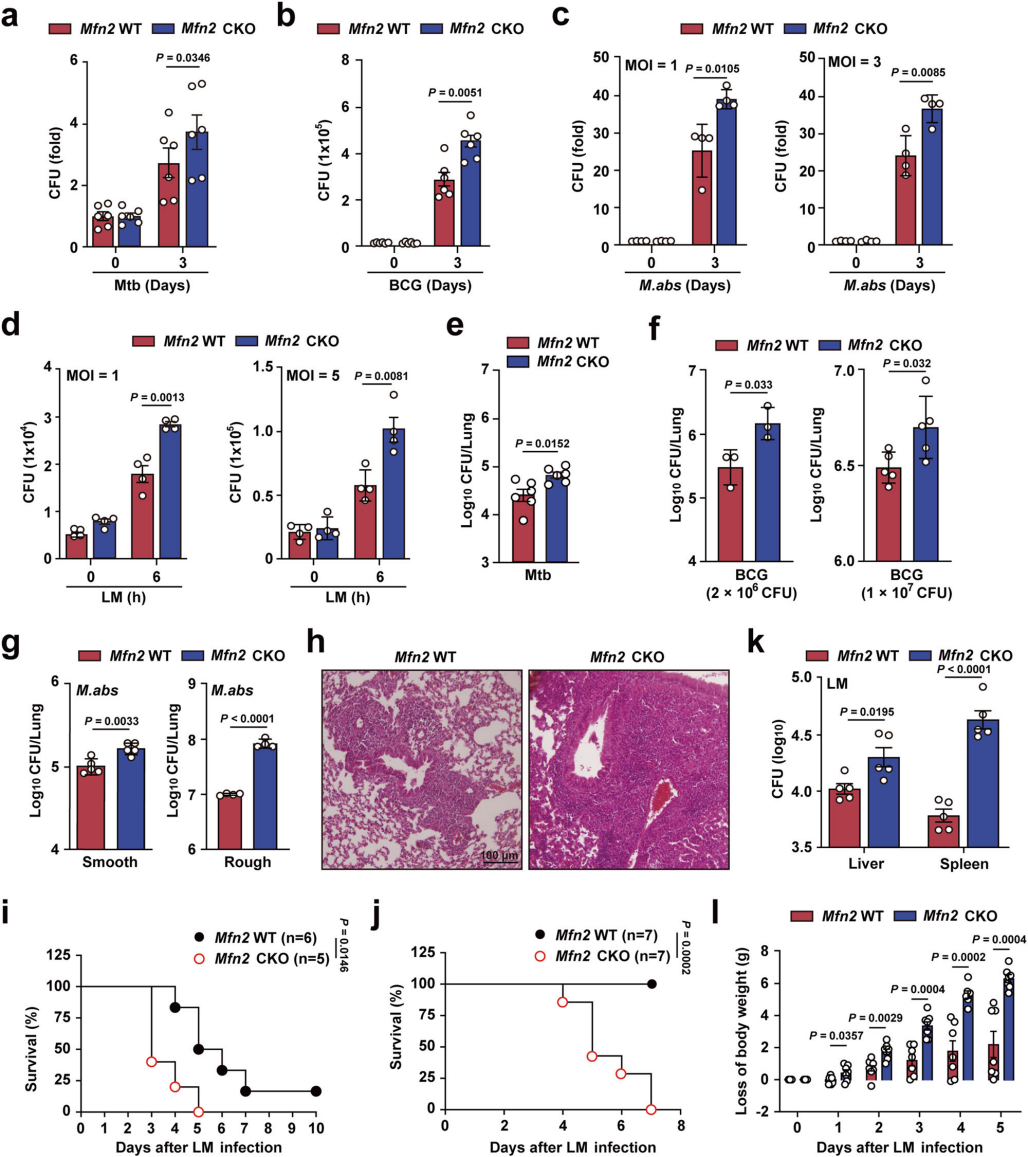
MFN2 is essential for antimicrobial responses during bacterial infection. **a**–**d** Intracellular survival assay after Mtb (MOI 3; *n* = 6) (**a**), BCG (MOI 3; *n* = 6) (**b**), *M. abscessus* (*M.abs*)-smooth (MOI 1 and 3; *n* = 4) (**c**), and LM (MOI 1 and 5; *n* = 4) (**d**) infection assessed in *Mfn2* WT and *Mfn2* CKO BMDMs after indicated time duration. **e**–**g** In vivo bacterial load in lung tissues from Mtb (**e**; *n* = 6, 5 × 10^4^ CFU), BCG (**f**; 2 × 10^6^ CFU, *n* = 3 and 1 × 10^7^ CFU, *n* = 5) at 7 days post infection (dpi) or *M.abs* (**g**; smooth *n* = 5, 2 × 10^6^ CFU and rough *n* = 4, 1 × 10^7^ CFU)-infected *Mfn2* WT and *Mfn2* CKO mice**. h** H&E staining of the BCG-infected lung tissue from *Mfn2* WT and *Mfn2* CKO mice. Representative images are shown. Scale bars, 100 μm. **i**, **j** Survival rate of *Mfn2* WT and *Mfn2* CKO mice infected with LM i.v. (WT *n* = 6, CKO *n* = 5) (**i**) and i.p. (*n* = 7) (**j**) monitored for indicated time. **k** In vivo bacterial load in liver and spleen from *Mfn2* WT and *Mfn2* CKO mice infected with LM (i.p.) for 72 h (*n* = 5). **l** Daily change in body weight of *Mfn2* WT and *Mfn2* CKO mice after LM infection. Loss of body weight is calculated subtracting the body weight of each day to that of day 0 (*n* = 7). Mean ± SEM are shown (**a**–**g**, **k**, **l**). Two-tailed Student’s *t* tests (**a**–**g**, **k**, **l**) and log-rank (Mantel–Cox) test (**i**, **j**) were used to measure the significance.

To investigate the role of MFN2 in myeloid cells in vitro and in vivo, we bred *Mfn2* CKO mice by crossing *Mfn2*^*flox/flox*^ mice with mice expressing Cre recombinase from the endogenous *lysozyme M* locus. To confirm the KO status of MFN2 in myeloid cells, we checked the expression of MFN2 in several organs and bone marrow-derived macrophages (BMDMs). Results confirmed that MFN2 is absent in BMDMs but is present in other types of tissues (Supplementary Fig. 1a). There were no significant differences in the phagocytic abilities between *Mfn2*^*f/f*^;*LysM Cre*^*–*^ (*Mfn2* WT) and *Mfn2* CKO BMDMs (Supplementary Fig. 1b). We then determined the physiological function of MFN2 in macrophages during mycobacterial and listerial infection. During infection with Mtb, *M. bovis* BCG (BCG), and *M. abscessus*, macrophages from *Mfn2* CKO mice had a higher capacity to replicate, with higher levels of intracellular colony forming units (CFUs) at different multiplicities of infection (MOIs), as compared to BMDMs from *Mfn2* WT mice (Fig. 1a–c). Similarly, the intracellular growth of *Listeria monocytogenes* (LM) was significantly higher in BMDMs from *Mfn2* CKO mice compared to BMDMs from *Mfn2* WT mice at different MOIs (Fig. 1d).

To further investigate the role of MFN2 in myeloid cells in vivo, we challenged *Mfn2* WT and *Mfn2* CKO mice with Mtb, BCG, or *M. abscessus* by intranasal instillation and monitored in vivo bacterial loads. The CFU counts from the lungs were significantly higher in the *Mfn2* CKO mice, as compared to WT littermate control mice (Fig. 1e–g). In addition, the number of granulomatous lung lesions was significantly increased in *Mfn2* CKO mice compared to *Mfn2* WT mice after infection with BCG (Fig. 1h). We next assessed whether MFN2 expression affected the host defense against LM intravenous (i.v.) or intraperitoneal (i.p.) infection. *Mfn2* CKO mice showed increased mortality after in vivo challenge with LM infection compared to *Mfn2* WT mice (Fig. 1i, j). *Mfn2* CKO mice exhibited significantly higher bacterial burden in the liver and spleen when compared to *Mfn2* WT mice (Fig. 1k). In addition, greater weight loss was observed in *Mfn2* CKO mice than in *Mfn2* WT mice after LM infection (Fig. 1l). These data confirmed that MFN2 plays an essential role in antimicrobial host defenses against intracellular bacterial infection.

### MFN2 deficiency impairs inflammatory responses during intracellular bacterial infection

**Fig. 2:**
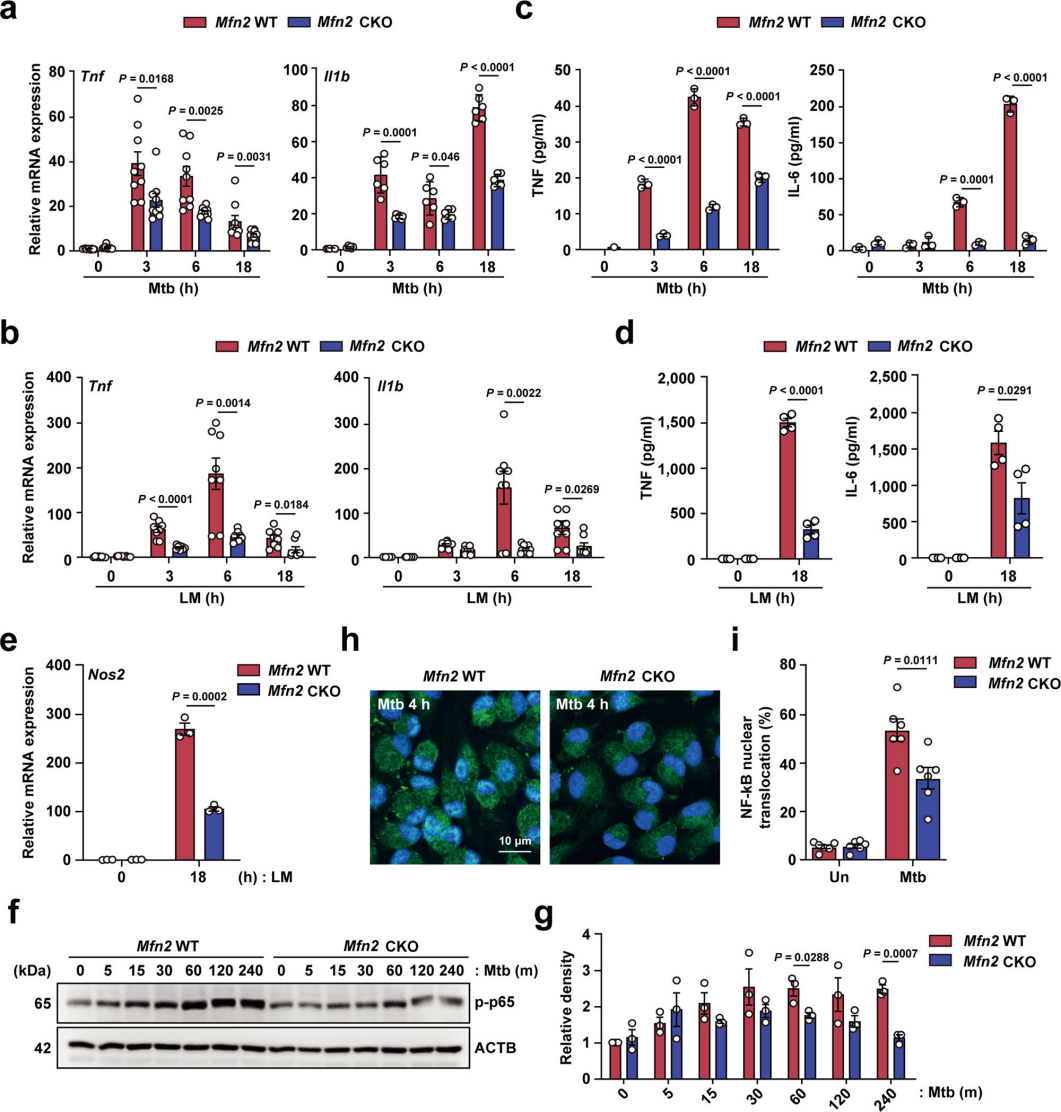
MFN2 is required for inflammatory reaction against bacterial infection. **a**, **b** qPCR analysis of *Tnf*, *Il1b* mRNA expression in *Mfn2* WT and *Mfn2* CKO BMDMs after Mtb (MOI 5) (**a**) or LM (MOI 5) (**b**) infection for indicated time (*n* = 6). **c**, **d** ELISA analysis of TNF and IL-6 in supernatant from Mtb (MOI 5) (**c**) or LM (MOI 5) (**d**) infected *Mfn2* WT or *Mfn2* CKO BMDMs. **e** qPCR analysis of *Nos2* after LM (MOI 5) infection for indicated time in PMs from *Mfn2* WT and *Mfn2* CKO mice (*n* = 3). **f** *Mfn2* WT and *Mfn2* CKO BMDMs infected with Mtb (MOI 5) for indicated time. Phospho-p65 (NF-κB) and ACTB levels were evaluated by western blot analysis. **g** Graph shows the densitometry analysis. **h** *Mfn2* WT and *Mfn2* CKO PMs were infected with Mtb (MOI 5) for 4 h and stained with anti-NF-κB p65 (green) and DAPI (for nuclei; blue). Representative immunofluorescence microscopy images. Scale bars, 10 μm. **i** Quantitation of p65 nuclear translocation. Fifty cells in six fields were counted in each group from two different experiments. Data are pooled from three (**a**, **b**) or representative of two (**h**) or three independent (**c**–**f**) experiments and are presented as mean ± SEM (**a**, **b**, **d**, **g**, **i**) or mean ± SD (**c**, **e**). Two-tailed Student’s *t* tests were used to measure the significance. Un, uninfected.

We next investigated the role of MFN2 in the activation of inflammatory responses in BMDMs or peritoneal macrophages (PMs) from *Mfn2* WT and *Mfn2* CKO mice. In response to different intracellular bacteria, we observed significantly lower mRNA levels of inflammatory cytokines such as tumor necrosis factor (TNF)-α and interleukin (IL)-1β in BMDMs from *Mfn2* CKO mice than in littermate controls (Fig. 2a for Mtb; Fig. 2b for LM; Supplementary Fig. 2a for BCG). ELISA analysis showed that TNF and IL-6 production was significantly inhibited in BMDMs from *Mfn2* CKO mice after Mtb or LM infection (Fig. 2c, d). In addition, iNOS (*Nos2*) mRNA expression was significantly lower in PMs from *Mfn2* CKO mice than in *Mfn2* WT PMs during infection with LM (Fig. 2e). Similarly, mRNA levels of *Tnf*, *Il1b*, *Il12p40*, and *Il6* were significantly lower in PMs from *Mfn2* CKO mice infected with Mtb or LM compared to those in PMs from *Mfn2* WT mice (Supplementary Fig. 2b, c). Moreover, treatment of BMDMs with mdivi-1, the general inhibitor of mitochondrial fragmentation^14,15^, decreased inflammatory cytokine generation (Supplementary Fig. 2d), suggesting a unique role for MFN2 in the regulation of macrophage inflammatory responses during infection.

We further examined whether MFN2 expression affected nuclear factor (NF)-κB activation. The results showed that the phosphorylation level of NF-κB p65 was lower in BMDMs (Fig. 2f, g) and PMs (Supplementary Fig. 2e) from *Mfn2* CKO mice compared to macrophages from *Mfn2* WT mice during Mtb infection. A similar finding was observed in LM-infected BMDMs from *Mfn2* CKO mice, compared with those from *Mfn2* WT mice (Supplementary Fig. 2f). In addition, Mtb-mediated nuclear translocation of NF-κB p65 was significantly lower in *Mfn2* CKO PMs compared to PMs from *Mfn2* WT mice (Fig. 2h, i). Together, these data demonstrate that macrophage MFN2 is required for the activation of inflammatory responses upon Mtb, BCG, or LM infection.

### MFN2 is required for the maintenance of aerobic glycolysis and the expression of *Ldha* and *Hif1a* in macrophages during infection

**Fig. 3:**
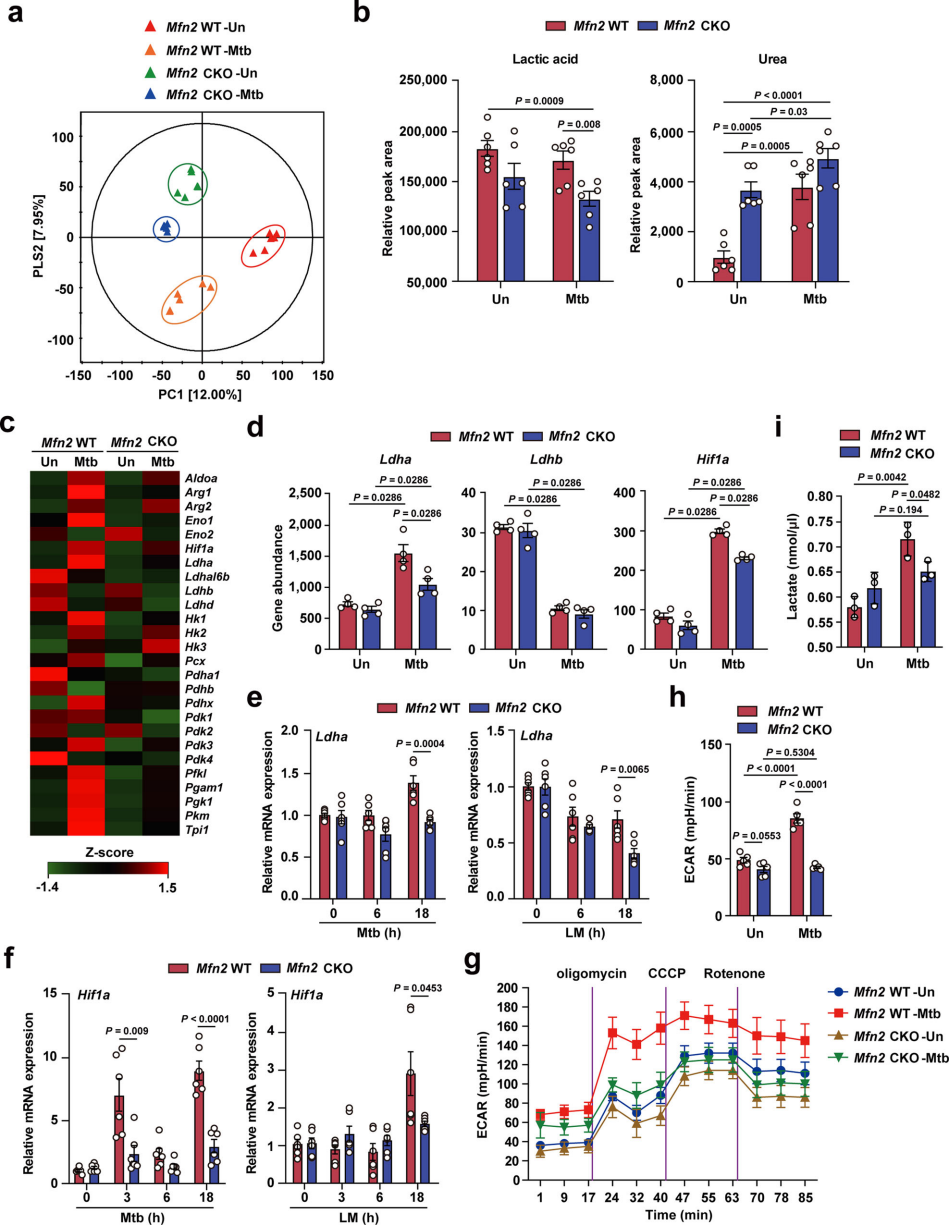
MFN2 is required for the induction of *Hif1α* and *Ldha* in macrophages. **a** PLS-DA score plots for metabolites in *Mfn2* WT and *Mfn2* CKO BMDMs between uninfected and after Mtb infection (MOI 5) based on GC-TOF/MS (VIP > 0.7, *p* value <.05). Each group designate with a different color (red: untreated *Mfn2* WT, orange: Mtb-infected *Mfn2* WT, green: untreated *Mfn2* CKO and blue: Mtb-infected *Mfn2* CKO). **b** The bar plots showed relative concentration of identified organic acids that was calculated by relative peak area from GC-TOF/MS analysis. **c** Heatmap analysis shows the differentially expressed genes in *Mfn2* WT and *Mfn2* CKO BMDMs before and after infection of Mtb for 18 h. The *z*-scores were calculated for each gene row using the average gene abundances of biological replicates. **d** Bar graph shows the abundance of genes *Ldha, Ldhb*, and *Hif1a* between *Mfn2* WT and *Mfn2* CKO BMDMs (*n* = 4). **e**, **f** qPCR analysis of *Ldha* (**e**) and *Hif1a* (**f**) mRNA expression in Mtb (MOI 5) and LM (MOI 5) infected *Mfn2* WT or *Mfn2* CKO BMDMs (*n* = 6). **g**, **h** Extracellular acidification profile (**g**) and representative glycolysis capacity parameter (**h**, *n* = 5) in *Mfn2* WT and *Mfn2* CKO PMs infected with Mtb (MOI 5) for 18 h. **i** Extracellular lactate level in *Mfn2* WT and *Mfn2* CKO BMDMs after Mtb (MOI 5) infection for 18 h. Data are representative or pooled from three independent experiments and are presented as mean ± SEM. The data were analyzed by the multiple *t* test with Holm–Sidak correction (**b**) or by two-tailed Student’s *t* test (**d**–**f**, **h**, **i**). Un, uninfected.

Accumulating evidence demonstrates that immune metabolic remodeling is critical for guiding the functional responses of numerous immune cells, including macrophages^16–18^. To further examine immunometabolic profiles in macrophages according to the *Mfn2* genotype and infectious pathogen, we performed GC-TOF/MS-based metabolomic analysis in *Mfn2* WT and *Mfn2* CKO BMDMs during Mtb infection. Partial least squares-discriminant analysis (PLS-DA) was used to visualize the clustering of each sample (Fig. 3a); the results showed that both *Mfn2* deficiency and Mtb infection significantly affected the metabolomic profiles of BMDMs (*p* < 0.05).

To reveal the major variables among the four experimental groups, variable importance in projection (VIP) scores (>0.7) of PLS-DA were used. A total of 23 metabolites in BMDMs including organic acids, amino acids, sugars, and sugar alcohols were identified as significantly discriminant metabolites. The relative concentrations of each metabolite were calculated and the heatmap showed different patterns of all classes among groups depending on MFN2 and/or Mtb infection (Supplementary Fig. 3a). Notably, lactic acid levels were significantly lower in BMDMs from *Mfn2* CKO mice after Mtb infection than in those from *Mfn2* WT mice (Fig. 3b, lactic acid panel). Although urea levels were significantly higher in uninfected *Mfn2* CKO BMDMs than in *Mfn2* WT BMDMs, no significant differences were detected between both cells after Mtb infection (Fig. 3b, urea panel). In addition, levels of other metabolites (succinic acid, itaconic acid, glycine, 5-oxo-proline, and saccharide 2) were not affected by *Mfn2* deficiency (Supplementary Fig. 3b).

To further address how MFN2 contributes to the maintenance of aerobic glycolysis and inflammatory responses in macrophages, we performed RNA-seq analysis. Global gene-expression analysis revealed the profiles of differentially expressed genes involved in metabolic reprogramming between *Mfn2* WT and *Mfn2* CKO BMDMs during Mtb infection (Fig. 3c, d). During glycolytic switch, HIF-1α acts as an important metabolic target, and lactate dehydrogenase isoform A (LDHA) is a downstream target of HIF-1α^19^. In particular, we noticed that the genes of *Ldha* and *Hif1a*, but not *Ldhb*, were upregulated in *Mfn2* WT BMDMs compared with those in *Mfn2* CKO BMDMs (Fig. 3e, f and Supplementary Fig. 3c) or PMs (Supplementary Fig. 3e, f) after Mtb infection. During LM infection, *Ldha*, *Ldhb*, and *Hif1a* levels were significantly lower in *Mfn2* CKO BMDMs (Fig. 3e, f and Supplementary Fig. 3d) and PMs (Supplementary Fig. 3f, g). In addition, the protein levels of HIF-α and LDHA were decreased in *Mfn2* CKO BMDMs, as compared to *Mfn2* WT BMDMs, after Mtb infection (Supplementary Fig. 3h). To confirm further the involvement of MFN2 in driving aerobic glycolysis during infection, extracellular acidification rate (ECAR), an indicator of enhanced glycolytic metabolism^20,21^, was measured in PMs. Both the ECAR and the glycolysis capacity parameter determined from ECAR were upregulated by Mtb infection in *Mfn2* WT PMs, but were significantly lower in *Mfn2* CKO PMs (Fig. 3g, h). Furthermore, extracellular lactate levels were significantly downregulated in *Mfn2* CKO BMDMs, compared with *Mfn2* WT BMDMs, after Mtb infection (Fig. 3i). These results reinforce the critical role of the macrophage MFN2 in optimal induction of aerobic glycolysis through the *Hif1a–Ldha* pathway upon bacterial infection.

### MFN2 is required for mitochondrial respiratory complex I and mitochondrial ROS (mtROS) generation in macrophages during infection

**Fig. 4:**
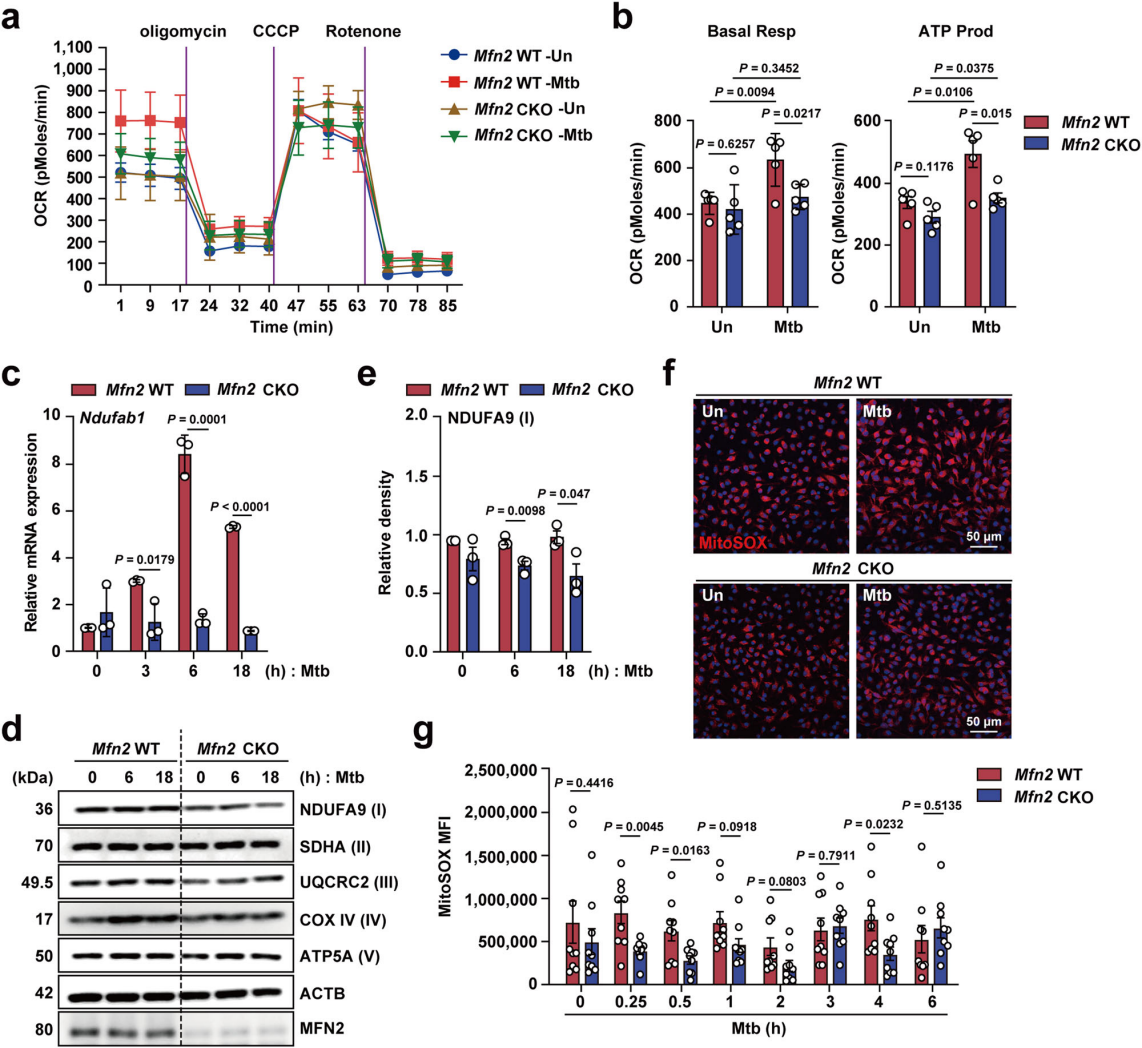
MFN2 is required for basal mitochondrial respiration and mtROS production during bacterial infection. **a**, **b** Respiratory profile (OCR) (**a**) and their respiratory parameters; Basal respiration (Basal resp) and ATP production OCR (ATP prod) (**b**) in *Mfn2* WT and *Mfn2* CKO PMs infected with Mtb (MOI 5) for 18 h. **c** qPCR analysis of *Ndufab1* in *Mfn2* WT and *Mfn2* CKO PMs infected with Mtb (MOI 5) for indicated time (*n* = 3). **d** Western blot analysis of OXPHOS complexes in *Mfn2* WT and *Mfn2* CKO BMDMs infected with Mtb (MOI 5) as indicated. **e** Densitometry analysis of NDUFA9 western blot represented in (**d**) (*n* = 3). **f** Representative MitoSOX (red) or DAPI (for nuclei; blue)-stained images. BMDMs from *Mfn2* WT or *Mfn2* CKO mice were infected with Mtb (MOI 5 for 4 h) and visualized by confocal microscopy. Scale bars, 50 μm. **g** Average mean fluorescence intensities (MFI) of MitoSOX. Data are presented as mean ± SD (**c**) or mean ± SEM (**b**, **e**, **g**) and are representative of three independent experiments. Two-tailed Student’s *t* test is used to calculate the significance (**b**, **c**, **e**, **g**). Un, uninfected.

Given that MFN2 is involved in the regulation of aerobic glycolysis, we next questioned whether oxidative phosphorylation differed between *Mfn2* WT and *Mfn2* CKO macrophages after infection. We analyzed mitochondrial respiration by measuring the oxygen consumption rate (OCR) in PMs from *Mfn2* WT and *Mfn2* CKO mice (Fig. 4a). We found that basal respiration and ATP production were significantly higher in *Mfn2* WT PMs than in *Mfn2* CKO PMs after Mtb infection (Fig. 4a, b). However, there were no differences in maximal respiration, non-mitochondrial respiration, and spare respiratory capacity, between *Mfn2* WT and *Mfn2* CKO PMs after Mtb infection (Supplementary Fig. 4a). We next measured the mRNA and protein levels of five components of the mitochondrial respiratory chain complexes. Notably, mRNA and protein levels of mitochondrial respiratory chain complex I (*Ndufab1*) were significantly inhibited in *Mfn2* CKO macrophages compared to those in *Mfn2* WT macrophages after Mtb infection (Fig. 4c and Supplementary Fig. 4c for PMs; Fig. 4d, e and Supplementary Fig. 4b for BMDMs). However, the levels of other mitochondrial respiratory chain complexes, including II (SDHA), III (UQCRC2), IV (COX4), and V (ATP5A1), did not differ significantly between *Mfn2* WT and *Mfn2* CKO BMDMs or PMs (Fig. 4d and Supplementary Fig. 4b, c).

Mitochondrial respiratory complex I is involved in the generation of mtROS^22,23^. In addition, mitochondrial oxidative stress results in the activation of HIF-1α expression and stabilization^24,25^. Therefore, we explored whether mtROS generation differs between *Mfn2* WT and *Mfn2* CKO BMDMs during infection. In *Mfn2* WT BMDMs, mtROS generation was upregulated by Mtb infection in a bimodal pattern, at 0.25–0.5 h and 4 h after Mtb infection (Fig. 4f, g). Importantly, mtROS production was significantly decreased in *Mfn2* CKO BMDMs at several time points (0.25–0.5 h and 4 h) after Mtb infection, when compared to those in *Mfn2* WT BMDMs (Fig. 4f, g). Collectively, these data suggest that MFN2 is required for mitochondrial respiratory complex I and mtROS production in macrophages during infection.

### MFN2-mediated mtROS production leads to the induction of HIF-1α, thereby promoting inflammatory signaling in macrophages during infection

**Fig. 5:**
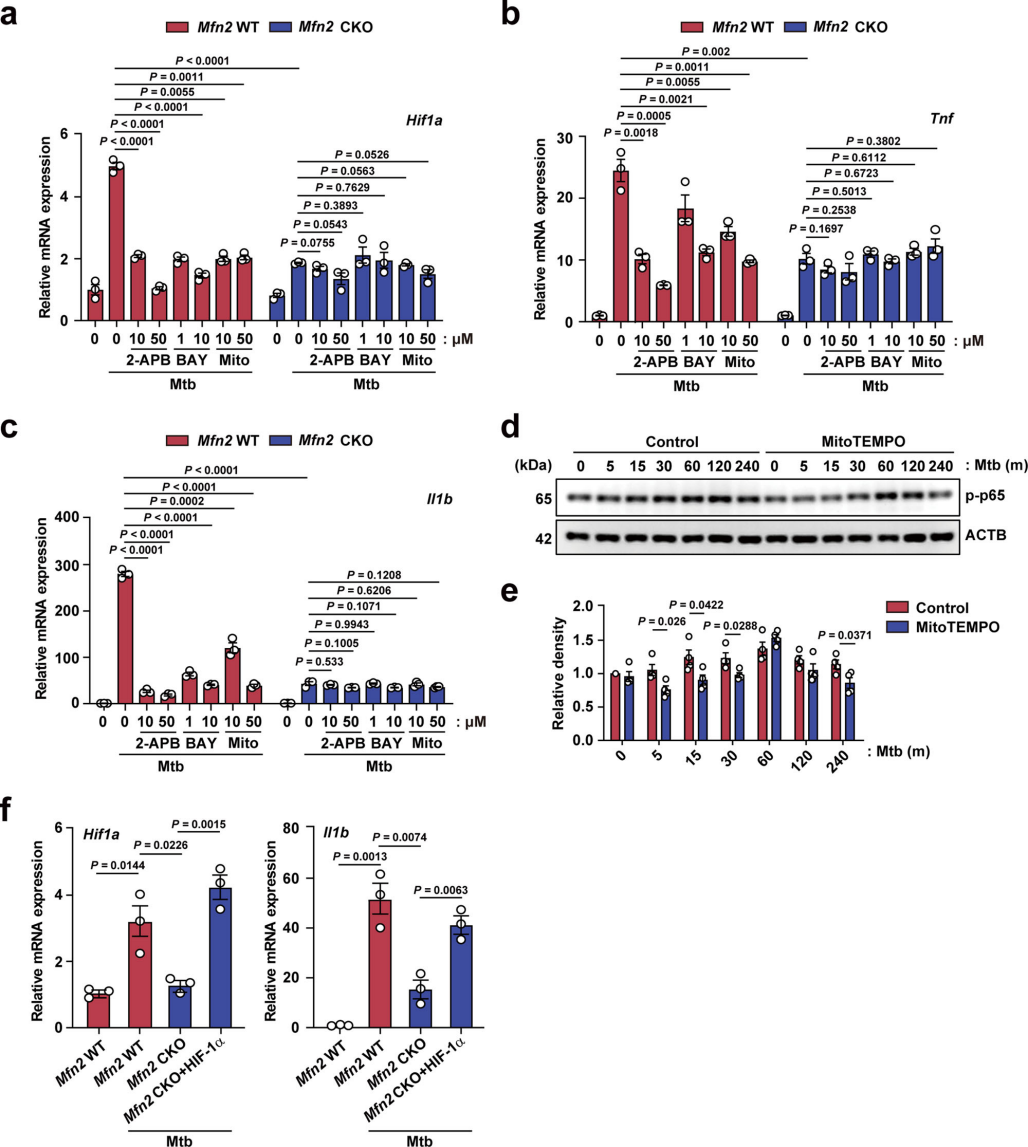
MFN2-mediated mtROS production control the HIF-1α expression and inflammatory signaling. **a**–**c** BMDMs from *Mfn2* WT or *Mfn2* CKO mice were infected with Mtb (MOI 5) and treated with indicated doses of 2-APB, BAY 87-2243 (BAY) or MitoTEMPO (Mito) for 18 h. qPCR was performed to detect the mRNA expression of *Hif1a* (**a**)*, Tnf* (**b**), and *Il1b* (**c**). **d** Western blot analysis of phosphorylation of p65 in *Mfn2* WT BMDMs treated with MitoTEMPO (100 μM) for 1 h and infected with Mtb (MOI 5) for indicated time. **e** Relative density is shown in graph (*n* = 4). **f** qPCR analysis of indicated genes in *Mfn2* WT or *Mfn2* CKO BMDMs transduced with lentivirus expressing HIF-1α for the overexpression and infected with Mtb (MOI 5) for 18 h (*n* = 3). Representative data of two to three independent experiments are shown. Two-tailed Student’s *t* test is used to calculate the significance (**a**–**c**, **e**, **f**). BAY, BAY 87-2243; Mito, MitoTEMPO.

Previous studies reported that oxidative stresses contribute to the induction of HIF-1α in vitro and in vivo^24,25^. We thus determined whether MFN2-mediated mtROS regulated the induction of HIF-1α and inflammatory signaling in macrophages. Interestingly, we found that mtROS scavenging by MitoTEMPO treatment significantly downregulated Mtb-induced *Hif1a*, *Tnf*, and *Il1b* mRNA expression (Fig. 5a–c) as well as NF-κB phosphorylation in *Mfn2* WT BMDMs (Fig. 5d, e). It was noted that HIF-1α blockade using BAY 87-2243 led to inhibition of *Tnf and Il1b* induced by Mtb (Fig. 5a–c), suggesting that MFN2-mediated HIF-1α is essentially required for inflammatory cytokine generation. In addition, 2-aminoethoxydiphenyl borate (2-APB), a cell-permeable modulator of Ins (1,4,5) P3-induced Ca^2+^ release, significantly downregulated Mtb-induced *Hif1a, Tnf*, and *Il1b* mRNA expression in *Mfn2* WT BMDMs (Fig. 5a–c). However, the regulatory effects of these pharmacological inhibitors for mtROS, HIF-1α, and Ins (1,4,5) P3-induced Ca^2+^ signaling were markedly attenuated in *Mfn2* CKO BMDMs (Fig. 5a–c). These data strongly suggest that MFN2-dependent *Hif1a* expression is dependent on Ins (1,4,5) P3-mediated Ca^2+^ signaling and mtROS production in macrophages and that mtROS-HIF-1α axis is crucial for inflammatory cytokine responses during infection.

We further questioned whether lentiviral vector-mediated overexpression of HIF-1α rescued inflammatory cytokine generation in *Mfn2* CKO macrophages during Mtb infection. As shown in Fig. 5f, Mtb-mediated *Il1b* mRNA expression was completely reversed by reconstitution of *Mfn2* CKO macrophages with HIF-1α. Taken together, these data strongly suggest that MFN2-mediated induction of HIF-1α is essential for the activation of inflammatory signaling in macrophages during infection.

### MFN2 deficiency leads to excessive mitochondrial fragmentation, but does not impact mitophagy activation in macrophages

**Fig. 6:**
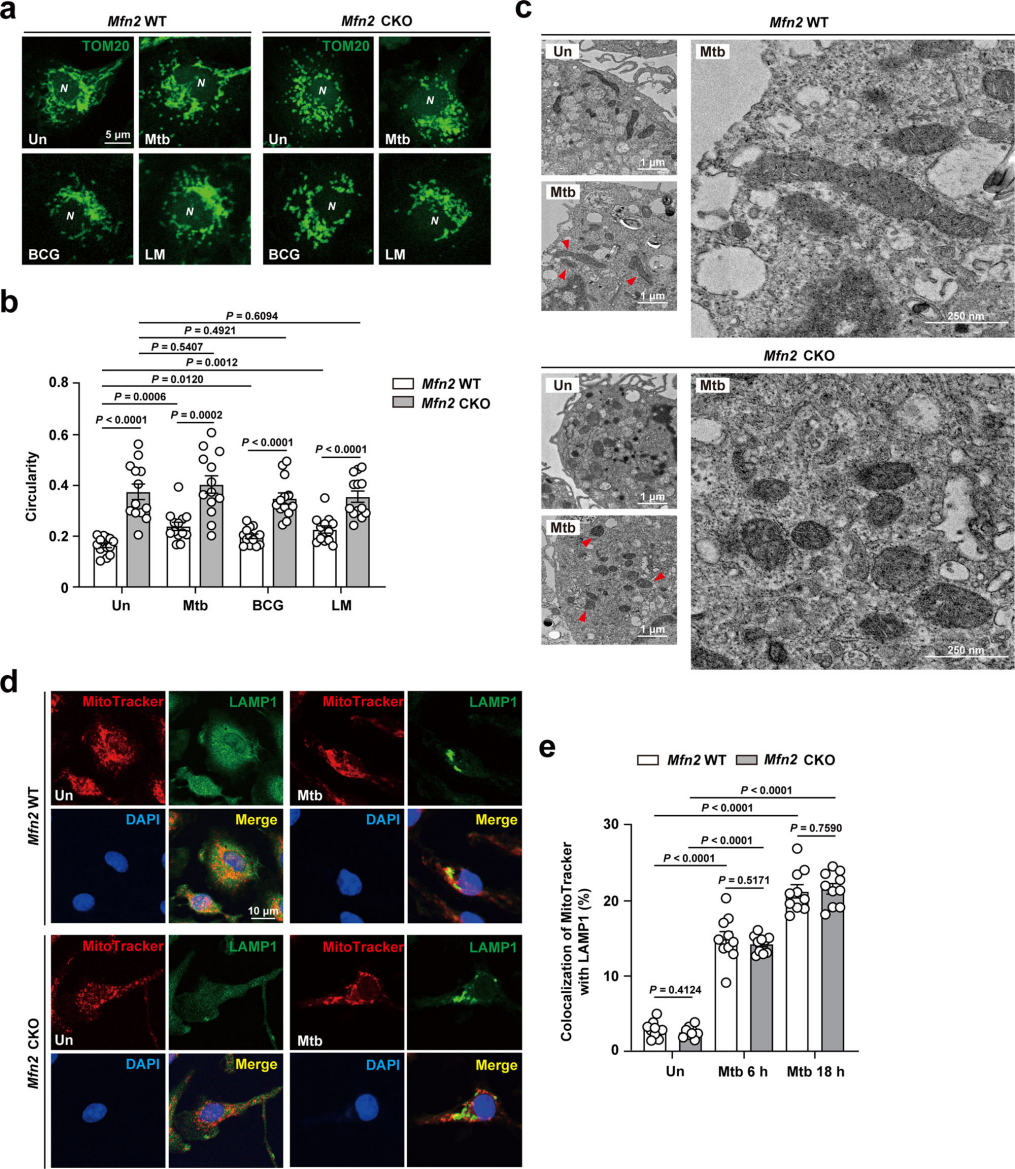
MFN2 controls excessive induction of mitochondrial fragmentation, but does not impact mitophagy, in macrophages during infection. **a** TOM20 (green) stained BMDMs from *Mfn2* WT and *Mfn2* CKO infected with Mtb (MOI 5 for 18 h), BCG (MOI 5 for 18 h), or LM (MOI 5 for 6 h). Cells were visualized by confocal microscopy. Scale bars, 5 μm. **b** Quantification of the mitochondrial morphology (circularity) in (**a**). **c** Representative electron microscopy image of *Mfn2* WT and *Mfn2* CKO BMDMs infected with Mtb (MOI 5) for 18 h. Scale bars, 1 μm and 250 nm. **d** BMDMs from *Mfn2* WT or *Mfn2* CKO mice were infected with Mtb (MOI 5 for 18 h) and stained with MitoTracker (red), LAMP1 (green), and DAPI (for nuclei; blue). Representative images. Scale bars, 10 μm. **e** Quantitative data of co-localization of MitoTracker and LAMP1 are shown. Fifty cells in 13 fields were counted in each group from two different experiments. Two-tailed Student’s *t* test is used to calculate the significance (**b**, **e**). Data are presented as mean ± SEM and are representative of three independent experiments. Un, uninfected; N, nucleus.

To gain further insight into the mechanism of antimicrobial defense by MFN2, we next compared mitochondrial dynamics between *Mfn2* WT and *Mfn2* CKO BMDMs during infection. Confocal imaging and ultrastructural analysis showed that various infectious stimuli (Mtb, BCG, and LM infection) induced a greater increase in mitochondrial fragmentation in *Mfn2* CKO BMDMs than in *Mfn2* WT BMDMs (Fig. 6a–c).

A recent study showed that LM robustly induces host mitophagy to impact antimicrobial host defense in macrophages^26^. We therefore compared mitophagy induction between *Mfn2* WT and *Mfn2* CKO BMDMs following LM or Mtb infection. Although LM infection robustly decreased the levels of TIM23, a mitochondrial inner membrane protein^27^, in both *Mfn2* WT and *Mfn2* CKO BMDMs, no significant difference in TIM23 intensity was detected between *Mfn2* WT and *Mfn2* CKO BMDMs before and after LM infection (Supplementary Fig. 5a, b). Consistent with our LM infection findings, TIM23 intensity was similar between *Mfn2* WT and *Mfn2* CKO BMDMs after 6 and 18 h post Mtb infection (Supplementary Fig. 6a, b). Furthermore, we found that there was no difference in co-localization of MitoTracker and LAMP1 between *Mfn2* WT and *Mfn2* CKO BMDMs after Mtb (Fig. 6d, e) or LM infection (Supplementary Fig. 5c, d). In addition, similar levels of co-localization of mitochondria and LC3 were observed between *Mfn2* WT and *Mfn2* CKO BMDMs following infection (Supplementary Fig. 5e for LM, Supplementary Fig. 6c for Mtb). Together, these results indicate that MFN2 is involved in the prevention of excessive mitochondrial fragmentation during infection. Although we did not analyze the differences in mitophagy flux between *Mfn2* WT and *Mfn2* CKO macrophages, our data strongly suggest that MFN2 does not affect mitophagy activity during intracellular bacterial infection.

### MFN2 promotes xenophagy activation against Mtb infection

**Fig. 7:**
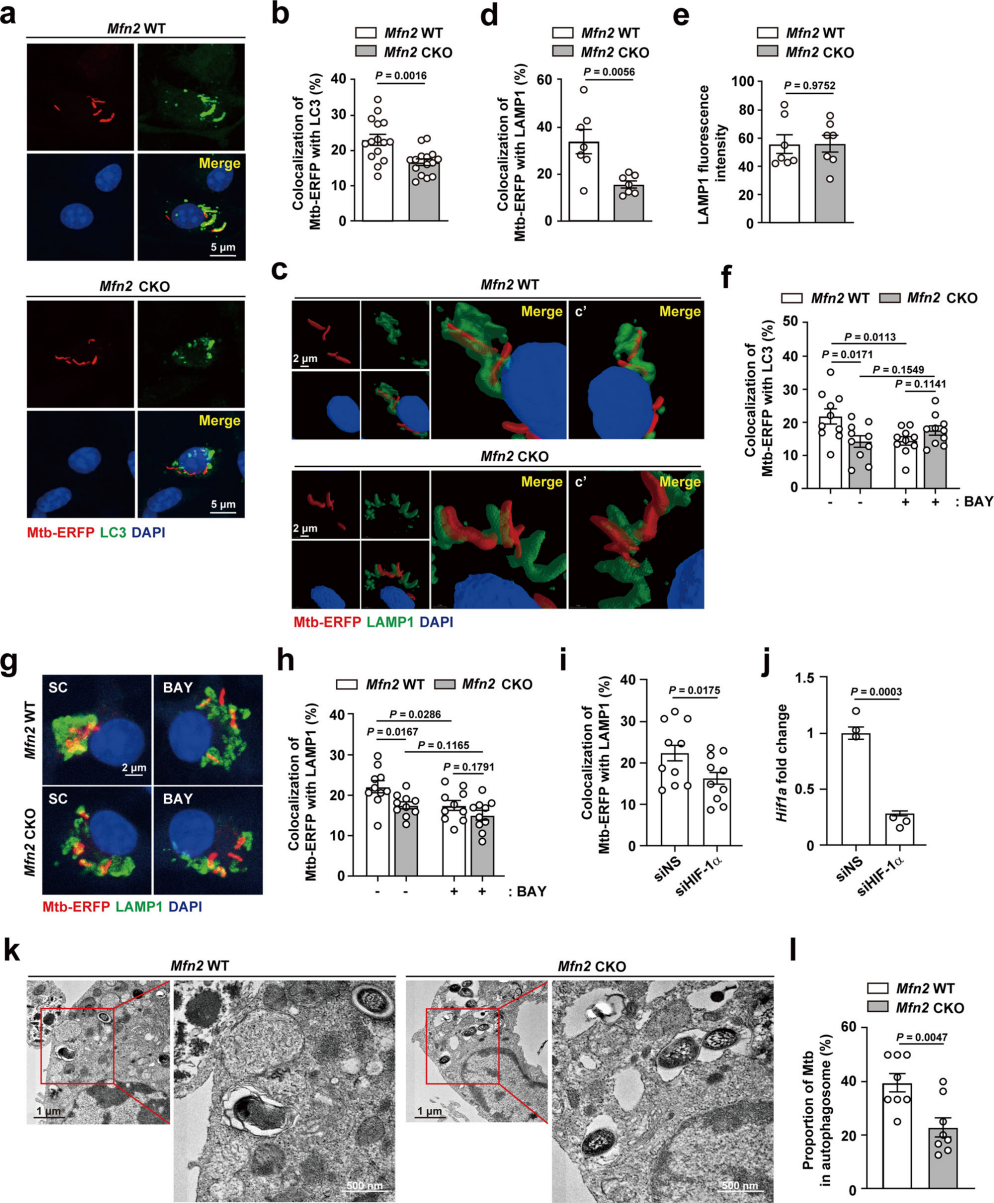
MFN2 promotes xenophagy activation during bacterial infection through HIF-1α. **a**, **b** *Mfn2* WT and *Mfn2* CKO BMDMs were infected with Mtb-ERFP (MOI 5) for 6 h. Cells were stained with LC3 (green) and DAPI (for nuclei; blue). **a** Cells were visualized by confocal microscopy. Scale bars, 5 μm. **b** Quantitative data of co-localization of Mtb-ERFP and LC3. Fifty cells in 15 fields were counted in each group from two different experiments. **c**–**e** BMDMs from *Mfn2* WT and *Mfn2* CKO mice infected with Mtb-ERFP (MOI 5) for 6 h and stained with LAMP1 (green) and DAPI (for nuclei; blue). **c** Confocal *z*-stack images were obtained and reconstituted to three dimensions. Scale bars, 2 μm. c’ shows different angles of the models. **d** Quantitative data of co-localization of Mtb-ERFP and LAMP1. Fifty cells in seven fields were counted in each group from two different experiments. **e** Quantitative data of LAMP1 intensity. Fifty cells in seven fields were counted in each group from two different experiments. **f**–**h** BMDMs from *Mfn2* WT and *Mfn2* CKO treated with BAY 87-2243 (BAY; 1 μM) for 1 h and infected with Mtb-ERFP (MOI 5) for 6 h. **f** Quantitative data of co-localization of Mtb-ERFP and LC3. Fifty cells in 15 fields were counted in each group from two different experiments. **g** Cells were stained with LAMP1 (green) and DAPI (for nuclei; blue). Representative images for Mtb-ERFP co-localization with LAMP1. Scale bars, 2 μm. **h** Quantitative data of co-localization of Mtb-ERFP and LAMP1. **i**, **j** RAW264.7 cells transfected with siNS (nonspecific siRNA) or siHIF-1α (siRNA targeting HIF-1α) and infected with Mtb-ERFP (MOI 5) for 6 h were stained with LAMP1 and DAPI (for nuclei). Quantitative data of co-localization of Mtb-ERFP and LAMP1 (**i**) and qPCR analysis performed to assess transfection efficiency (**j**). **k** Representative electron microscopy image of *Mfn2* WT and *Mfn2* CKO BMDMs infected with Mtb (MOI 5). Scale bars, 1 μm and 500 nm. **l** Quantitative data of autophagosomes containing Mtb. Data are presented as mean ± SEM. Two-tailed Student’s *t* test is used to calculate the significance (**b**, **d**–**f**, **h**–**j**, **l**). SC, solvent control; BAY, BAY 87-2243.

Given that MFN2 plays an essential role in the fusion of autophagosomes and lysosomes in cardiomyocytes^28^, we further investigated whether MFN2 is involved in the regulation of xenophagy during infection. We performed image analysis of Mtb phagosomes, autophagosomes, and lysosomes to assess whether co-localization efficiency depends on MFN2 expression in macrophages. We observed that co-localization between Mtb phagosomes and LC3 autophagosomes was significantly downregulated in *Mfn2* CKO BMDMs, compared with those in *Mfn2* WT BMDMs (Fig. 7a, b). In addition, MFN2 deficiency led to a significant decrease in the level of co-localization between Mtb phagosomes and lysosomes after infection (Fig. 7c, d). Furthermore, we measured the autophagy flux by using a retroviral vector containing mCherry-enhanced green fluorescent protein (EGFP)-LC3B^29,30^ between *Mfn2* WT and *Mfn2* CKO BMDMs. It was noted that autophagosome (yellow dots) formation was increased, whereas the autolysosome (red dots) formation was decreased in *Mfn2* CKO BMDMs, compared to those in *Mfn2* WT BMDMs, during Mtb infection (Supplementary Fig. 7a, b). In addition, bafilomycin A1 treatment increased the LC3 punctate formation in *Mfn2* WT BMDMs, but not in *Mfn2* CKO BMDMs, during Mtb infection (Supplementary Fig. 7c). These data strongly suggest that MFN2 is essential for autophagic flux in macrophages during Mtb infection. However, we detected no significant difference in LAMP1 expression levels between *Mfn2* WT and *Mfn2* CKO BMDMs during Mtb infection (Fig. 7e).

Previous studies have reported that HIF-1α is involved in the activation of autophagic proteolysis and autophagic flux in several types of cells^31–33^. We therefore examined whether HIF-1α contributes to xenophagy activation during Mtb infection. HIF-1α blockade by pharmacological inhibitor BAY 87-2243 led to significant inhibition of co-localization of Mtb phagosomes with autophagosomes and lysosomes in *Mfn2* WT BMDMs, but had no effect in *Mfn2* CKO BMDMs (Fig. 7f–h). Also, knocking-down of HIF-1α using small interfering RNAs (siRNA) specific to HIF1-α led to significant inhibition of Mtb co-localization with LAMP1 in RAW264.7 cells (Fig. 7i). Knockdown efficiency of the siRNA is shown in Fig. 7j. Furthermore, TEM analysis revealed the reduced Mtb in autophagosomal structures in *Mfn2* CKO BMDMs, when compared to those in *Mfn2* WT BMDMs (Fig. 7k, l). Together, these data suggest that MFN2-HIF-1α is essentially required for the activation of xenophagy against Mtb in macrophages.

### MFN2 interaction with Rab7 is required for xenophagy activation in macrophages during Mtb infection

**Fig. 8:**
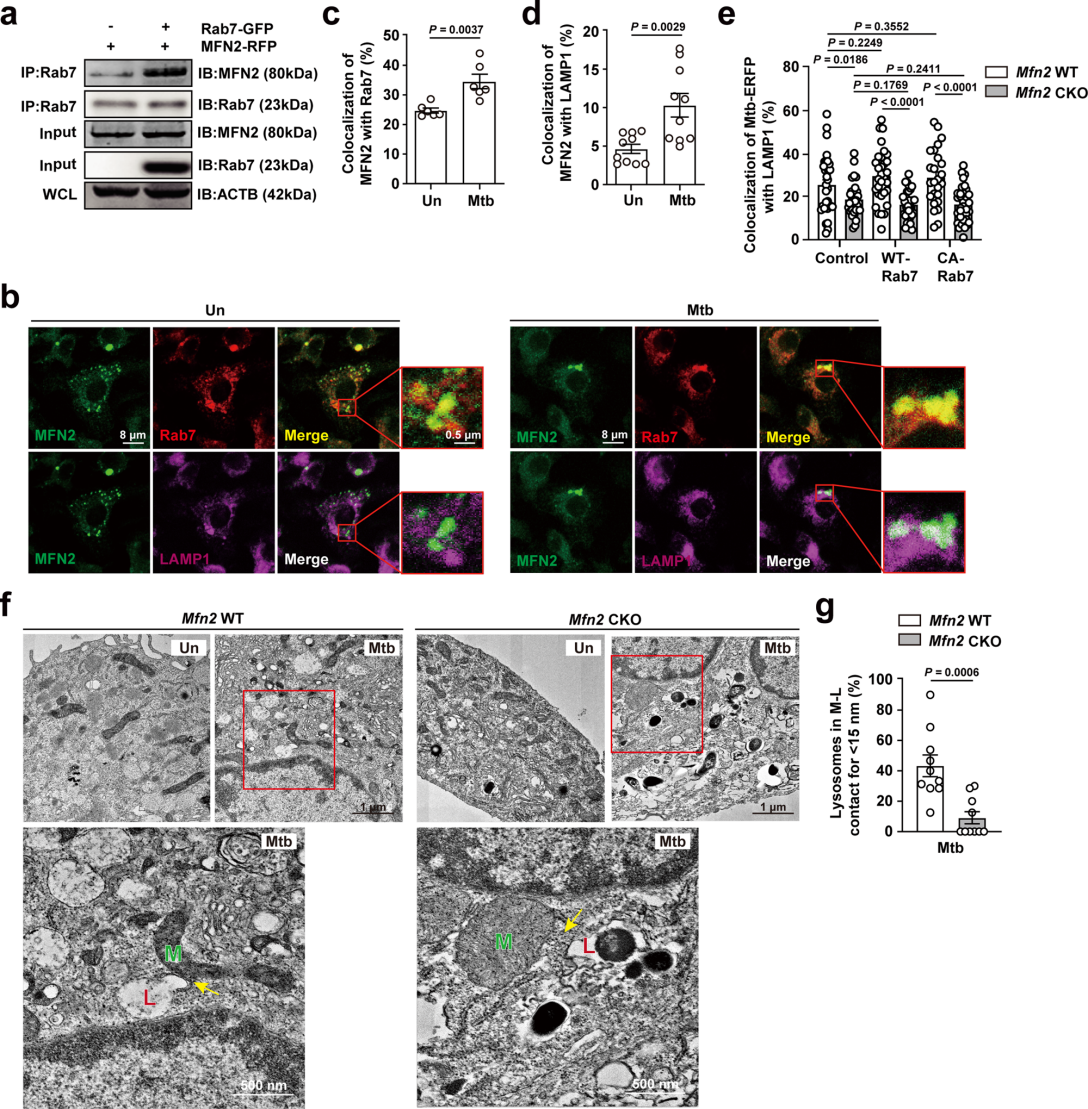
MFN2 interacts with Rab7 for the activation of xenophagy. **a** Immunoprecipation analysis from RAW264.7 cells transfected with MFN2-RFP or Rab7-GFP. Representative immunoblots for the indicated protein expression. **b**–**d** Confocal microscopy images of BMDMs infected with Mtb (MOI 5) and stained with MFN2 (green), Rab7 (red), and LAMP1 (magenta). Representative images for MFN2 co-localization with Rab7 or LAMP1. Scale bars, 8 μm and 0.5 μm (**b**). **c**, **d** Quantitative data of co-localization of MFN2 and Rab7 (**c**) or LAMP1 (**d**). Fifty cells in six (**c**) or ten (**d**) fields were counted in each group from two different experiments. **e** BMDMs from *Mfn2* WT and *Mfn2* CKO mice transduced with a control lentivirus, virus expressing a mouse WT-Rab7 or constitutively active (CA)-Rab7 plasmid for 48 h, were infected with Mtb-ERFP (MOI 5) for 6 h and then stained with LAMP1 and DAPI (for nuclei). Quantitative data of co-localization of Mtb-ERFP and LAMP1 are shown. **f**, **g** BMDMs from *Mfn2* WT and *Mfn2* CKO were infected with Mtb (MOI 5) for 18 h. Representative electron microscopy image of mitochondria (M) and lysosome (L) contact (yellow arrows) (**f**). Scale bars, 1 μm and 500 nm. Quantitative data of percentage of lysosomes contacting mitochondria (for <15 nm; **g**). Data are presented as mean ± SEM. Two-tailed Student’s *t* test is used to calculate the significance (**c**–**e**, **g**). WCL, whole cell lysate; CA, constitutively active; Un, uninfected; M, mitochondria; L, lysosome.

Previous studies have shown that MFN2 interacts with the small GTPase Rab7 to promote autophagosome–lysosome fusion in cardiomyocytes^28^. We therefore evaluated the association of MFN2 with late-endosomal protein Rab7 and lysosomal protein LAMP1 in macrophages in the context of infection. Co-immunoprecipitation analysis revealed that Rab7 and MFN2 interacted together in RAW264.7 cells (Fig. 8a). Confocal analysis also showed significant upregulation of MFN2 interactions with Rab7 in BMDMs after Mtb infection (Fig. 8b, c). Also, the interaction between MFN2 and LAMP1 was significantly increased as analyzed by confocal microscopy in macrophages after Mtb infection (Fig. 8d). We then assessed whether MFN2-Rab7 interaction contributed to the activation of xenophagy in macrophages. To examine this, we overexpressed *Mfn2* CKO BMDMs with lentiviral vectors containing Rab7-WT or Rab7-CA (constitutive active form) and measured the xenophagy activation in these cells. As shown in Fig. 8e, we found that either Rab7-WT or Rab7-CA overexpression failed to rescue the decreased co-localization between Mtb and LAMP1 in *Mfn2* CKO BMDMs. These data suggest that Rab7 alone under MFN2 deficiency is insufficient to activate xenophagy during Mtb infection.

MFN2 is recognized to play an essential role in ER–mitochondrial tethering and calcium signaling through inter-organelle association^34,35^. However, whether MFN2 is involved in the connection between mitochondrial and lysosomal compartments remains unknown. We found that Mtb infection robustly upregulated co-localization between mitochondrial outer membrane protein TOM20 and lysosomal protein LAMP1 in *Mfn2* WT BMDMs, and that this upregulation was significantly inhibited in *Mfn2* CKO BMDMs (Supplementary Fig. 8a, b). TEM analysis showed a marked increase in contact between mitochondrial and lysosomal compartments in *Mfn2* WT BMDMs after Mtb infection, which was dramatically reduced in *Mfn2* CKO BMDMs (Fig. 8f, g). These data strongly suggest that MFN2 is required for tethering mitochondrial and late-endosomal/lysosomal compartments during infection.

## Discussion

The coordinated control of fusion and fission in mitochondrial dynamics is essential for maintaining mitochondrial function^1,3,36^. However, the roles and mechanisms by which key modulators operating mitochondrial dynamics remain poorly understood in terms of innate host defenses during intracellular bacterial infection. Our data suggest two overarching considerations regarding the mechanisms by which MFN2 contributes to innate host defense through coordination of immunometabolism and xenophagy via HIF-1α during intracellular bacterial infection. First, MFN2 is involved in the control of immunometabolism, which is required to activate inflammation in macrophages via HIF-1α-mediated aerobic glycolysis. Second, MFN2 contributes to the activation of xenophagy through HIF-1α, thereby enhancing phagosomal maturation and antimicrobial responses during Mtb infection.

We found that myeloid cell-specific MFN2 is essential for antimicrobial and inflammatory responses in vitro and in vivo during intracellular bacterial infection. These data partly correlate with a recent study reporting the protective role for myeloid MFN2 in innate immune responses during sepsis and bacterial infection^12^. Although the previous study showed that MFN2 deficiency results in a decreased phagocytosis in macrophages^12^, we did not observe any difference of Mtb phagocytosis between *Mfn2* WT and *Mfn2* CKO macrophages. The discrepancy might be due to different receptor–ligand interactions in macrophages between previous studies^12^ and ours. Our data are also partly consistent with previous findings that MFN2 was required for IL-1β secretion during infection with RNA viruses^11^. MFN2 interacted with mitochondrial antiviral signaling (MAVS) to suppress antiviral immunity through impaired production of interferons (IFNs) and inflammatory cytokines^37,38^. Recently, epigoitrin treatment inhibited MFN2 expression, thus activating MAVS and antiviral cytokine IFN-β during influenza virus infection^39^. Moreover, previous studies have shown that MFN2 is involved in the immunomodulatory effect of ghrelin, an orexigenic hormone, in the production of IL-12 in response to LPS^40^. The accumulated data may support the key function of MFN2 in the regulation of inflammatory responses that are necessary for pathogen clearance during bacterial and viral infection.

Our study provides evidence that MFN2 is required for the activation of inflammatory responses and NF-κB signaling by maintaining aerobic glycolysis and HIF-1α induction in macrophages. HIF-1α is a master regulator of aerobic glycolysis and plays a critical role in cell reprogramming and macrophage polarization to the M1 phenotype^41,42^. Importantly, we found that MFN2 mediated a pathogen-induced increase in the basal mitochondrial respiration, ATP production, and generation of mtROS, which were involved in HIF-1α expression and inflammatory signaling in macrophages. These data are partly consistent with previous findings that oxidative stresses contribute to HIF-1α induction^24,25^. Notably, our data showed that MFN2 is crucial for the activation of mitochondrial respiratory complex I, which is a major contributor to the generation of mtROS^22,23^. As there was a significant increase in urea in MFN2 deficiency, we examined the shift to M2 phenotypes of BMDMs and PMs from *Mfn2* CKO mice. Indeed, the arginase-1 mRNA level was significantly increased in *Mfn2* CKO cells, compared to *Mfn2* WT cells (see Fig. 3c, RNA-seq analysis). Therefore, our work suggests that MFN2 functions in the suppression of macrophage differentiation into the M2 phenotype as well as driving aerobic glycolysis and inflammatory responses through HIF-1α during infection. The role of MFN2 in maintaining aerobic glycolysis seems to be unique in the settings of infection and inflammation, because there was no significant difference in glycolysis between unstimulated/uninfected *Mfn2* WT and *Mfn2* CKO macrophages^12^. In addition, MFN1/2 depletion was shown to promote the glycolytic metabolic reprogramming in mouse embryonic fibroblasts^19^, suggesting that MFN2 controls intracellular metabolism in a context-dependent manner.

We found that MFN2 plays a role in controlling excessive mitochondrial fragmentation during intracellular bacterial infection. Although mitochondrial fragmentation was upregulated in MFN2 deficiency, mitophagy activities were comparable between BMDMs from *Mfn2* WT and *Mfn2* CKO mice. Both mitophagy and xenophagy were activated in macrophages during intracellular bacterial infection, with a certain degree of overlap. Our data are important to show that MFN2 is critically required for xenophagy activation, but not mitophagy, during infection. Notably, our data suggest that HIF-1α is a key element for MFN2-mediated innate host defense through the induction of xenophagy as well as aerobic glycolysis and inflammation, during Mtb infection. Our data are partly consistent with recent studies that HIF-1α is involved in the activation of autophagic proteolysis and autophagic flux in various cells^31–33^. Our data also demonstrate that MFN2 interacts with Rab7, a principal molecule involved in phagosomal maturation^43–45^ during Mtb infection. These data partly agree with previous findings that MFN2 plays a role in cardiac autophagy through interaction with the small GTPase Rab7^28^. In addition, our data showed that reconstitution of *Mfn2* CKO macrophages with either Rab7-WT or -CA failed to rescue the co-localization of Mtb and LAMP1 in these cells. These data strongly suggest that MFN2-Rab7 interaction is, at least, required for maintaining the basal level of xenophagy activation during infection. Future studies are needed to clarify the exact underlying molecular and biochemical mechanisms through which MFN2-Rab7 crosstalk contributes to innate host defense during infection.

**Fig. 9:**
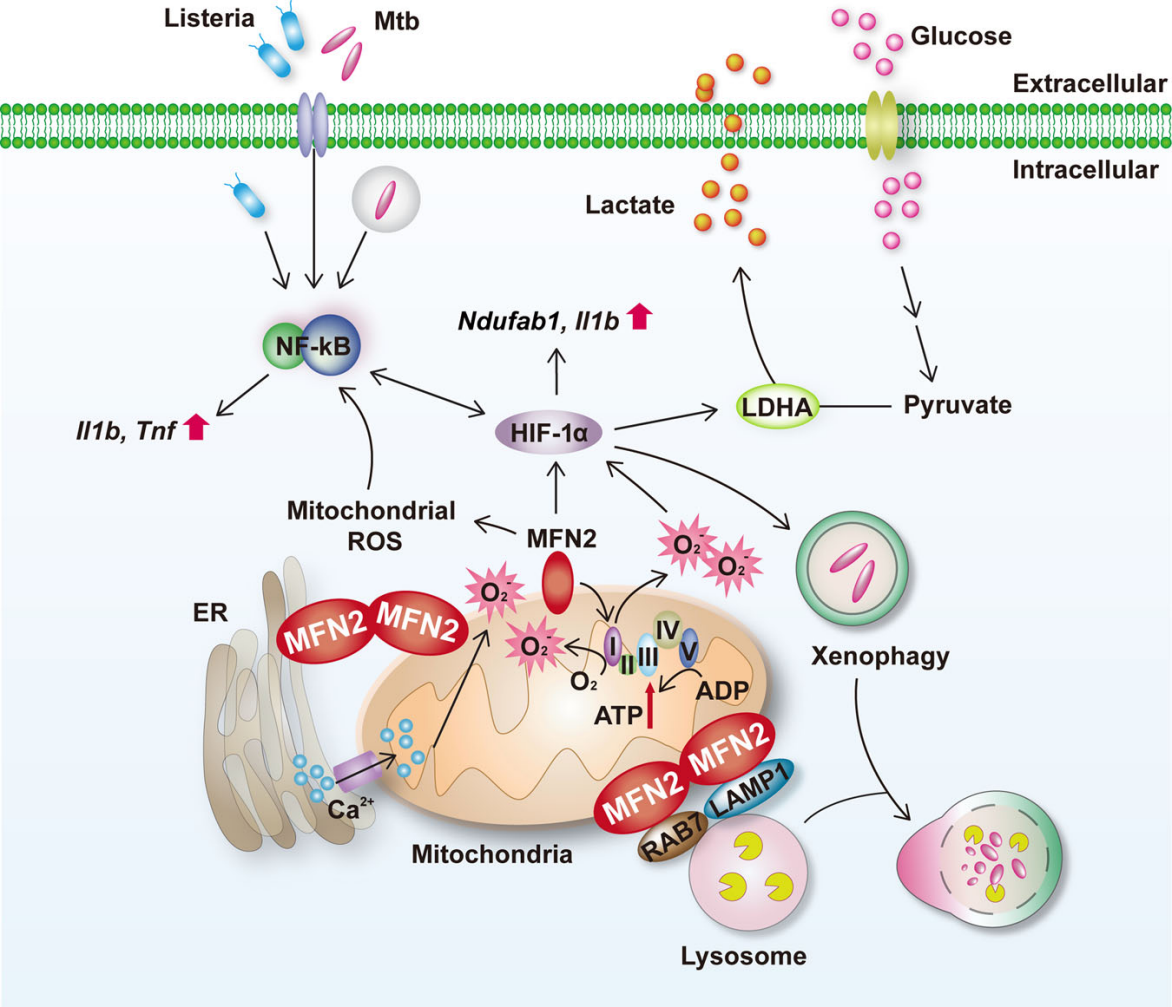
A proposed model of MFN2-mediated regulation of innate immune responses during bacterial infection. In response to mycobacterial or listerial infection, MFN2 is essentially required for antimicrobial host defense through immunometabolic remodeling and xenophagy activation. MFN2 contributes to the activation of inflammatory responses via the maintenance of aerobic glycolysis. During infection, MFN2 is crucial for the basal respiration of mitochondria and generation of mitochondrial ROS, which results in the HIF-1α expression and inflammatory signaling. In addition to immunometabolic regulation, MFN2 plays a role in the enhancement of xenophagic activation through interactions with Rab7.

Inter-organelle communication, particularly mitochondrial–lysosomal interaction, is critically involved in various biological functions including the regulation of mitochondrial dynamics, metabolite transfer, and cellular homeostasis^46,47^. The key mediating molecule(s) in the connection between mitochondrial and lysosomal compartments remain largely unknown. In addition to the known role of MFN2 in ER–mitochondrial tethering and calcium signaling through inter-organelle crosstalk^34,35^, our data demonstrate that MFN2 is a key mediator of contact between mitochondria and lysosomes during Mtb infection. We propose that MFN2-mediated contact between mitochondria and late-endosomal/lysosomal compartments may contribute to antimicrobial host defense during Mtb infection. As summarized visually in Fig. 9, this study identified previously unappreciated mechanisms by which MFN2 promotes innate host defenses through coordinated orchestration of immunometabolism, xenophagy, and mitochondrial–lysosomal contacts, during intracellular bacterial infection.

## Materials and methods

### Mice

The mice used in individual experiments were age- (6–8 weeks) and sex-matched. *Mfn2*^fl/fl^ mice were purchased from The Jackson Laboratory. *LysM*-Cre mice were kindly provided by Dr. C.-H. Lee (Korea Research Institute of Bioscience and Biotechnology). Mice were maintained under specific pathogen-free conditions. All animal-related procedures were reviewed and approved by the Institutional Animal Care and Use Committee, Chungnam National University School of Medicine, Daejeon, Korea (CNU-00944).

### Cells

Primary BMDMs, PMs, and the murine macrophage cell line RAW264.7 (ATCC, TIB-71) were cultured in Dulbecco’s modified Eagle’s medium (DMEM; Lonza) containing 10% fetal bovine serum (FBS; Lonza), penicillin (100 IU/ml), and streptomycin (100 μg/ml). Bone marrow cells from *Mfn2* WT or *Mfn2* CKO mice were cultured for 3–5 days in the presence of macrophage colony-stimulating factor (R&D Systems) for BMDMs differentiation. PMs were prepared as mentioned previously with slight modifications^48^. Briefly, each mouse was injected (i.p.) with 1 ml of 3% thioglycollate and after 3 days the peritoneal fluid was collected in ice-cold PBS containing 3% FBS. The collected cell suspension was centrifuged and the cells that were counted and cultured overnight, before proceeding to experiments. Phoenix AMPHO (ATCC, CRL-3213) cells were maintained in DMEM and incubated in a 37 °C humidified atmosphere with 5% CO_2_.

### Bacterial strain and culture

Mtb H37Rv was kindly provided by Dr. R. L. Friedman (University of Arizona). *M. bovis* BCG was obtained from the Korean Institute of Tuberculosis. Mtb, BCG, and smooth-morphotype *M. abscessus* (ATCC, 19977) were grown at 37 °C with shaking in Middlebrook 7H9 broth (Difco) supplemented with 0.5% glycerol, 0.05% Tween-80 (Sigma-Aldrich), and oleic albumin dextrose catalase (OADC; BD Biosciences). The isogenic rough type *M. abscessus* was obtained through continuous anaerobic passage. Rough type *M. abscessus* was separated into single cells using a tissue homogenizer consisting of a Teflon rod and a glass tube (Wheaton) for 2 min at 3000 rpm. For Mtb-expressing enhanced red fluorescent protein (ERFP) strains, Mtb-ERFP was grown in Middlebrook 7H9 medium supplemented with OADC and 50 μg/ml kanamycin (Sigma-Aldrich). All mycobacterial suspensions were aliquoted and stored at −80 °C. For all experiments, mid-log-phase bacteria (absorbance 0.4) were used. Representative vials were thawed and CFUs enumerated by serially diluting and plating on Middlebrook 7H10 agar (Difco).

LM (ATCC, BAA-679) was grown in brain heart infusion (BHI; LPS solutions) broth at 37 °C for 16 h. A 1:10 dilution of the bacterial culture was grown for 2 h at 37 °C prior to infection. Bacterial numbers were counted on a BHI agar plate.

### Bacterial infection

Cells were infected with indicated MOIs of mycobacteria or LM for 2 h or 30 min, respectively. Extracellular bacteria were washed with PBS and infected cells were further cultured in fresh medium for indicated time.

For in vivo infection, frozen bacteria were thawed and inoculated intranasally (Mtb; 5 × 10^4^ CFU/mice, BCG; 2 × 10^6^ or 1 x 10^7^ CFU/mice, *M. abscessus* smooth strain; 2 × 10^6^ CFU/mice or *M. abscessus* rough strain; 1 × 10^7^ CFU/mice). To measure the bacterial burden, lungs were harvested after sacrificing the mice after 1 day or 7 days of Mtb infection, 7 days of BCG and *M. abscessus* smooth strain infection, or after 5 days of *M. abscessus* rough stain infection. Tissue were homogenized in PBS and serial dilutions of the homogenates were plated in 7H10 agar plates. After 2–3 weeks of incubation, colonies formed in the plates were counted.

For LM infection, mice were injected i.v. (1 × 10^5^ CFU/mice) or i.p. (for in vivo bacterial load 1 × 10^5^, for survival study 0.75 × 10^5^ and for the measurement of body weight 0.5 × 10^5^ CFU/mice). To measure the bacterial loads in liver and spleen, mice were sacrificed 72 h after the infection, organs were homogenized in PBS, and serial dilutions of the homogenates were plated in BHI agar plates. The colonies formed were counted after 24 h. Mice weight were measured daily to assess the change in body weight. Results were calculated after subtracting the body weight each day to the weight before infection.

### CFU assay

For the assessment of intracellular bacterial viability, mycobacteria infected cells were lysed in distilled water to release the intracellular bacteria. The harvested bacteria were then plated in Middlebrook 7H10 agar and incubated for 3 weeks, and colonies were counted. In case of LM infection experiments, cells were lysed in distilled water and serial dilution of the lysate were plated in BHI agar plates. Colonies were counted after 24 h of incubation.

### Histology

Lungs from BCG-infected *Mfn2* WT or *Mfn2* CKO mice were harvested after 10 days post infection. Tissue samples were fixed in 10% formalin and then they were embedded in paraffin wax. Paraffin sections of 4 μm thickness were cut and were stained with hematoxylin and eosin for light microscopic examination.

### Antibodies and reagents

Following antibodies and reagents were purchased from indicated commercial sources. Anti-LC3A/B (PM036) was purchased from MBL International. 4′-6-Diamidino-2-phenylindole dihydrochloride (DAPI; D9542) and MitoTEMPO (SML0737) were purchased from Sigma-Aldrich. 2-APB (100065) and BAY 87-2243 (6980) were from Millipore and Tocris biosciences, respectively. Anti-TIM23 (1:400; 611223) was from BD Biosciences. Alexa Fluor 405-conjugated anti-rat IgG (1:400; A48261), Alexa Fluor 488-conjugated anti-rabbit IgG (1:400; A17041), Alexa Fluor 594-conjugated anti-rabbit IgG (1:400; A21207), Alexa Fluor 594-conjugated anti-goat IgG (1:400; A11058), MitoSOX Red (M36008), and anti-ATP5A (1:1000; 459240) were purchased from Invitrogen. Anti-Rab7 (1:400; sc-6563), anti-TOM20 (1:400; sc-11415), anti-LAMP1 (1:400; sc-19992), anti-ACTB (1:4000, sc-47778), and anti- NF-κB p65 (sc-8008 for immunofluorescence), anti-GAPDH (1:1000, sc-25778), and protein A/G agarose were purchased from Santa Cruz Biotechnology. Anti-phospho NF-κB (p65) (1:2000; 3033 for western blot), anti-SDHA (1:1000; 5839), anti-COX IV (1:2000; 4844), anti-MFN2 (1:1000 for western blot, 1:400 for immunofluorescence; 9482), and anti-LDHA (1:1000; 2012) were purchased from Cell Signaling; anti-NDUFA9 (1:1000; ab14713), anti-UQCRC2 (1:1000; ab14745) were from Abcam. Anti-HIF-1α antibody (1:1000, NB100-479) was purchased from Novus Biologicals. MitoTracker Deep Red FM (M22426) was purchased from Thermo Fisher Scientific. Mouse TNF (558534) and IL-6 (555240) ELISA kits were purchased from BD biosciences. Radio immunoprecipitation assay (RIPA) buffer was purchased from ELPIS biotech. Protease and phosphatase inhibitor cocktails were purchased from Roche. Lactate Colorimetric/Fluorometric Assay Kit (K607-100) was purchased from Biovision, Inc.

### Macrophage RNA-seq analysis

Total RNA from macrophage was isolated using TRIzol reagent (Invitrogen) and PureLink RNA Mini Kit (Invitrogen), according to the manufacturer’s instruction. Complementary DNA (cDNA) library preparation was conducted using the TruSeq Stranded Total RNA LT sample prep kit (Ribo-Zero Human/Mouse/Rat) according to the manufacturer’s instruction (Illumina). cDNA sequencing of 16 samples was performed by Illumina HiSeq 4000 in a 151 paired-end mode. We used the Snakemake workflow to process the raw reads^49^; FastQC (version 0.11.9) was used to preprocess and control quality of raw reads^50^; Cutadapt (version 2.1) was used to trim of adapter sequence^51^; Trimmed reads in FASTQ format were quantified at the transcript level using Salmon^52^ against an Ensemble reference mouse genome (GRCm38 March 2020). The results of transcripts quantification were aggregated to the gene level using tximport package (version 1.16)^53^ and differential expression of genes was analyzed using the DESeq2 package (version 1.28)^54^ on R program (version 3.6.3). Genes were considered as significantly differentially expressed if the adjusted *p* value (Benjamini–Hochberg multiple test correction method) was less than 0.05. Heatmap was generated with stats package (version 3.6.2) and the *z*-scores were calculated for each gene row using the average gene abundances of biological replicates on R program (version 3.6.3).

### Real-time quantitative PCR

Total RNA and cDNA synthesis was done as described above. Real-time PCR was performed using SYBR green master mix (Qiagen) and primers for indicated genes, in Rotor-Gene Q 2plex system (Qiagen). Data were analyzed using 2^ΔΔ^ threshold cycle (Ct) method where beta-actin was used for normalization. Data are expressed as relative fold changes. Primers sequences (mouse) were as follows: *Tnf* forward: 5’-CCCACGTCGTAGCAAACCAC-3’, reverse: 5’-GCAGCCTTGTCCCTTGAAGA-3’; *Il1b* forward: 5’-TGACGGACCCCAAAAGATGA-3’, reverse: 5’-AAAGACACAGGTAGCTGCCA-3’; *Il12p40* forward: 5’-AGGTCACACTGGACCAAAGG-3’, reverse: 5’-TGGTTTGATGATGTCCCTGA-3’; *Il6* forward: 5’-ACAAAGCCAGAGTCCTTCAGA-3’, reverse: 5’-TGGTCCTTAGCCACTCCTTC-3’; *Hif1a* forward: 5’-CAAGATCTCGGCGAAGCAA-3’, reverse: 5’-GGTGAGCCTCATAACAGAAGCTTT-3’; *Ldha* forward: 5’-TGCCTACGAGGTGATCAAGCT-3’, reverse: 5’-GCACCCGCCTAAGGTTCTTC-3’; *Ldhb* forward: 5’-AGTCTCCCGTGCATCCTCAA-3’, reverse: 5’-AGGGTGTCCGCACTCTTCCT-3’; *Nos2* forward: 5’-GACGAGACGGATAGGCAGAG-3’, reverse: 5’-GTGGGGTTGTTGCTGAACTT-3’; *Ndufab1* forward: 5’-GGACCGAGTTCTGTATGTCTTG-3’, reverse: 5’-AAACCCAAATTCGTCTTCCATG-3’; *Actin* forward: 5’-CCACCATGTACCCAGGCATT-3’, reverse: 5’-AGGGTGTAAAACGCAGCTCA-3’.

### Plasmid and small interfering RNA transfection

The DNA of *Rab7* (hMU001574) and *Mfn2* (KU024998) were purchased from Korea human gene bank and were cloned to peGFP-C3 (#6082-1) and pDsRed2 (#632404) vectors (Clontech Inc), respectively. Plasmids transfection was done using Lipofectamine 2000 (12566014, Invitrogen) according to the manufacturer’s instruction. Silencing of HIF-1α was achieved using siRNAs for mouse HIF-1α target sequences (sc-35562, Santa Cruz Biotechnology,) and negative control siRNAs (sc-37007, Santa Cruz Biotechnology). RAW264.7 cells were transfected with siRNA oligonucleotide using Lipofectamine 3000 (Invitrogen) according to the manufacturer’s instructions.

### Western blot and immunoprecipitation analysis

Cells were lysed in RIPA buffer supplemented with protease inhibitor cocktail and phosphatase inhibitor cocktails and equal amount of proteins were mixed with SDS sample buffer and boiled for 5–7 min. The samples were subjected to SDS-PAGE and then transferred on PVDF membrane. The membranes were blocked in 5% skim milk in Tris-buffered saline containing 0.1% Tween 20 (TBS-T) for 1 h at room temperature, and then incubated overnight with primary antibodies at 4 °C. Then the membranes were washed using TBS-T and further incubated with appropriate secondary antibodies for 1 h at room temperature. The immune-reactive proteins were detected using chemiluminescence kit. ImageJ software was used for densitometry analysis. For immunoprecipitation assay, cell lysates were incubated overnight with appropriate antibodies at 4 °C under gentle rotation and were precipitated using protein A/G agarose beads. The beads were mixed with 1X SDS sample buffer, boiled and were subjected to SDS-PAGE as explained above. The full, uncropped blot/gel images are shown in Supplementary Fig. 9.

### Image analysis

To quantify the phagosomal maturation, cells were visualized directly by confocal laser-scanning microscopy, and the images were captured using Leica software (LAS X; Leica). Quantification of mycobacterial co-localization with autophagosomes and lysosomes was performed by counting the red (non-co-localized) and yellow (co-localized) mycobacteria. Each experiment was completed on duplicate coverslips and the results are expressed as the mean and standard deviation. Images of dynamic cell co-localization were recorded as vertical *z*-stacks. LAS X small 2.0 and Adobe Photoshop 7 (Adobe Systems) were used for image processing. *Mfn2* WT or CKO BMDMs infected with Mtb or LM for indicated time were stained with MitoTracker Red, MFN2 (using Alexa Fluor 488-conjugated anti-rabbit IgG), Rab7 (using Alexa Fluor 594-conjugated anti-goat IgG), or LAMP1 (using Alexa Fluor 405-conjugated anti-rat IgG) to analyze the co-localization of MFN2 with Rab7 or LAMP1 and MitoTracker with LAMP1. BMDMs pretreated with Bafilomycin A1 (BafA1, 200 nM, B1793, Sigma-Aldrich) for 2 h and infected with Mtb-ERFP (MOI 5) for 6 h were stained with LC3 (green) and DAPI (for nuclei). Fluorescence images were acquired using a confocal laser-scanning microscopy (LAS X; Leica), with constant excitation, emission, pinhole, and exposure time parameters. The ImageJ software was used to analyze the percentage of lysosomes in contacts and co-localization, and LC3 intensity.

To quantify mitochondrial morphology, confocal images were processed in ImageJ software and analyzed with the publicly available ImageJ macro for mitochondrial morphology, Mito‐Morphology, designed by Ruben K. Dagda^55^. The macro returns the circularity value for each mitochondrion in the cell and the average circularity value of each measured cell.

### Measurement of mitochondrial ROS

Mtb-infected *Mfn2* WT and *Mfn2* CKO BMDMs were incubated with 2.5 μM MitoSOX^TM^ Red Mitochondrial Superoxide Indicator (Invitrogen). After 15 min, cells were washed and measured using confocal laser-scanning microscope (TCS SP8; Leica). To analyze data, the ImageJ software was used.

### Measurement of lactate

*Mfn2* WT or *Mfn2* CKO BMDMs were infected with Mtb for 18 h and supernatant was collected to measure the extracellular Lactate level using Lactate assay kit according to the manufacturer’s instructions.

### Production of lentivirus vector and lentivirus

Plasmids for lentivirus production were constructed by PCR amplification and Gibson Assembly Cloning (New England Biolabs). Both of pLenti-CMV-Rab7 WT and Rab7 CA form were generated by replacing the EGFP of pLenti-CMV-EGFP vector to CFP-Rab7 WT and CA fragments at the BamHI and EcoRI enzyme sites. CFP-Rab7 WT and CA form fragments were PCR-amplified from CMV-CFP-Rab7 WT and CA vectors, respectively. Primers used for PCR reaction were CFP-Rab7-F (5’-ggt cta tat aag cag agc tgg atc cgc cac cat ggt gag caa ggg cga gga g-3’) and CFP-Rab7-R (5’-gat tat cga taa gct tga tat cga att ctc agc aac tgc agc ttt ctg-3’). To generate the pLenti-EGFP-HIF-1α, PCR-amplified HIF-1α from HA-HIF--1α-pcDNA3 vector (addgene plasmid #18949) was inserted into pLenti-CMV-EGFP vector excised with AgeI and EcoRI restriction enzyme. HIF-1α was amplified through PCR reaction with HIF-1α-F (5’-cga gct gta caa gcc acc ggt aat gga ggg cgc cgg-3’) and HIF-1α-R (5’-gat tat cga taa gct tga tat cga att ctc agt taa ctt gat cca aag ctc tga gta att ctt cac-3’).

For lentivirus production, the lentivirus plasmid, Δ8.9, and VSV-G were co-introduced into HEK293T cells with PEI (Polyethylenimine) transfection reagent. Transfection solution was prepared by adding PEI reagent and DNA vectors to the Opti-MEM (Invitrogen) at a ratio of 2.5:1. After 72 h after transfection, the culture medium was collected and centrifuged at 626 × g for 5 min to remove cells. Then, the supernatants filtered through the filter with 0.45 μm pore (Millipore). Ultracentrifugation of filtrate at 107,000 × g for 2 h at 4 °C was carried out to concentrate lentivirus. The viral pellet was gently resuspended in ice-cold PBS and 5 μl aliquots were stored −80 °C refrigerator.

### Generation of a tandem LC3B retroviral vector

The production of a tandem LC3B retroviral vector (mCherry-EGFP-LC3B) for the measurement of autophagic flux was performed as described previously^56^. Briefly, Phoenix amphotropic cells were seeded into a six-well plate and co-transfected with 0.75 μg of packaging plasmid pCL-Eco (Addgene), 0.25 μg of envelope plasmid pMDG (Addgene), and 1 μg of pBABE puro mCherry-EGFP-LC3B plasmid (Addgene, 22418) using Lipofectamine 2000 (Invitrogen). After 6 h, the medium was replaced with fresh culture medium. The retrovirus containing medium was harvested at 24 and 48 h post transfection and filtered through a 0.45-μm syringe filter.

### Transmission electron microscopy

For transmission electron microscopy analysis, samples were sequentially fixed with 2.5 % glutaraldehyde and 1% osmium tetroxide on ice for 2 h and washed with PBS. The cells were then dehydrated in ethanol and propylene oxide series, embedded in Epon 812 mixture, and polymerized in an oven at 70 °C for 24 h. The sections acquired from polymerized blocks were collected on 150 mesh copper grids, counterstained with uranyl acetate and lead citrate, and examined with Bio-HVEM system (JEM-1400Plus at 120 kV and JEM-1000BEF at 1000 kV, JEOL).

### Immunofluorescence and confocal microscopy

Cells were cultured on coverslips and infected with Mtb, BCG, or LM as described above. After the appropriate infection, cells were washed three times with PBS, fixed with 4% paraformaldehyde for 15 min, permeabilized with 0.25% Triton X-100 (Sigma-Aldrich) for 10 min, and incubated with primary antibodies for 2 h at room temperature. Cells were washed with PBS to remove excess primary antibodies and then incubated with secondary antibodies for 1 h at room temperature. Nuclei were stained with DAPI for 5 min and fluorescence images were observed using a confocal laser-scanning microscope (TCS SP8; Leica).

### Flow cytometry

BMDMs were analyzed by flow cytometry for phagocytosis using a FACS Canto II flow cytometer, as indicated by the manufacturer (Becton Dickinson). BMDMs were infected with Mtb-ERFP and washed with PBS. Cells were assayed immediately and flow cytometry data were collected and analyzed using FlowJo software (Tree Star).

### ELISA

Cell supernatant after appropriate infection were collected and stored at −80 °C. ELISA was performed according to the protocol recommended by the manufacturer.

### OCR analysis in vitro

A Seahorse Bioscience XF24 analyzer (Seahorse Bioscience) was used to analyze the OCR and ECAR. The XF24 biosensor cartridge was activated with 1 ml of XF24 calibrant solution (Seahorse Bioscience) per well for 24 h at 37 °C in a non-CO_2_ incubation system. *Mfn2* WT and *Mfn2* CKO PMs were seeded at 5 × 10^5^ cells per well and incubated overnight at 37 °C were infected with Mtb (MOI 5) for 18 h. After the addition of 590 µl assay media in each well, the cell plate was incubated for 1 h at 37 °C in a non-CO_2_ incubation system. After measurement of basal OCR and ECAR, 20 µg/ml oligomycin (an ATPase inhibitor, final conc. 2 µg/ml), 50 µM CCCP (an uncoupler, final conc. 5 µM), and 20 µM rotenone (mitochondrial complex I inhibitor, final conc. 2 µM) were injected into each well. OCR and ECAR analysis were performed at 37 °C and data are presented as per total number of cells.

### Intracellular metabolite extraction

Cell samples were harvested and washed three times with PBS. Then 400 μl methanol (70%, v/v) was added to each sample to break up using a MM400 mixer mill (Retsch^®^) at a frequency of 30 Hz for 10 min, followed by 10 min of sonication at 4 °C (Hettich). Next, the extracts were centrifuged at 15,000 rpm for 10 min at 4 °C and supernatants were filtered using 0.2-μm polytetrafluoroethylene syringe filters. The filtered supernatants were dried using a speed-vacuum concentrator (Biotron).

### GC-TOF/MS analysis and data processing

The dried samples were processed to two steps of a derivatization reaction prior to GC-TOF/MS analysis by MetaMass (Seoul, Korea). Briefly, the oximation was conducted first by adding 35 μl of methoxyamine hydrochloride in pyridine (20 mg/ml) to the dried samples. The reaction mixture was incubated at 30 °C for 90 min. Subsequently, the silylation was performed by adding 35 μl of N-Methyl-N-(trimethylsilyl) trifluoroacetamide to the incubated reaction mixture, followed by a 37 °C incubation for 30 min. All the samples were filtered through a Millex GP 0.22-μm filter (Merck Millipore) prior to analysis. GC-TOF/MS analysis was performed using an Agilent 7890A system (Agilent Technologies) with Agilent 7693 autosampler and Pegasus HT TOF-MS (Leco Corporation). The GC-TOF/MS raw data were acquired by MetaMass (Seoul, Korea) using LECO Chroma TOFTM software (version 4.44, LECO Corp.) and raw data were converted into the NetCDF format (*.cdf). Using the Metalign software package (http://www.metalign.nl), peak detection, retention time correction, and alignment were processed and exported to Excel file (Microsoft Corp.). The multivariate statistical analyses were performed using SIMCA-P+ (version 12.0; Umetrics). PLS-DA modeling was performed to compare the different metabolites each experimental groups. The significantly discriminant variables among experimental groups were selected based on VIP values >0.7 and tested for significance at *p* value <0.05.

### Statistics and reproducibility

For graphs, all data were analyzed using GraphPad Prism (version 5.0 or 8.4.0). Differences were evaluated using a two-tailed Student’s *t* test. For in vivo survival experiments, statistical significance was evaluated using the log-rank (Mantel–Cox) test. Significant differences of PLS-DA were determined by analysis of variance using PASW Statistics 18 software (SPSS Inc.). The box plots were rendered using the relative peak area of unique metabolites masses by STATISTICA 7 software (StatSoft Inc) and the data were analyzed by multiple *t* test with Holm–Sidak correction for multiple comparisons using GraphPad Prism (version 8.0.2) for windows. All reported results were replicable. The number of animals and the number of replicates for each experiment are mentioned in figure legends.

### Reporting summary

Further information on research design is available in the Nature Research Reporting Summary linked to this article.

## Supplementary information

Supplementary Information

Descriptions of Additional Supplementary Files

Supplementary Data 1

Reporting Summary

## Data Availability

All data underlying the main and Supplementary figures are either available online in Supplementary Data 1, or available from the corresponding authors, upon reasonable request. All of the sequencing data that support the findings of the study have been deposited in the NCBI under accession code GSE169172.

## References

[1] Ni HM, Williams JA, Ding WX (2015). Mitochondrial dynamics and mitochondrial quality control. Redox Biol..

[2] Twig G (2008). Fission and selective fusion govern mitochondrial segregation and elimination by autophagy. EMBO J..

[3] Cerveny KL, Tamura Y, Zhang Z, Jensen RE, Sesaki H (2007). Regulation of mitochondrial fusion and division. Trends Cell Biol..

[4] Rovira-Llopis S (2017). Mitochondrial dynamics in type 2 diabetes: pathophysiological implications. Redox Biol..

[5] Calkins MJ, Manczak M, Mao P, Shirendeb U, Reddy PH (2011). Impaired mitochondrial biogenesis, defective axonal transport of mitochondria, abnormal mitochondrial dynamics and synaptic degeneration in a mouse model of Alzheimer’s disease. Hum. Mol. Genet.

[6] Song M, Mihara K, Chen Y, Scorrano L, Dorn GW (2015). Mitochondrial fission and fusion factors reciprocally orchestrate mitophagic culling in mouse hearts and cultured fibroblasts. Cell Metab..

[7] Song M, Dorn GW (2015). Mitoconfusion: noncanonical functioning of dynamism factors in static mitochondria of the heart. Cell Metab..

[8] Biel TG (2016). Sirtuin 1 suppresses mitochondrial dysfunction of ischemic mouse livers in a mitofusin 2-dependent manner. Cell Death Differ..

[9] Sebastian D (2012). Mitofusin 2 (Mfn2) links mitochondrial and endoplasmic reticulum function with insulin signaling and is essential for normal glucose homeostasis. Proc. Natl Acad. Sci. USA..

[10] Zorzano A, Hernandez-Alvarez MI, Sebastian D, Munoz JP (2015). Mitofusin 2 as a driver that controls energy metabolism and insulin signaling. Antioxid. Redox Signal.

[11] Ichinohe T, Yamazaki T, Koshiba T, Yanagi Y (2013). Mitochondrial protein mitofusin 2 is required for NLRP3 inflammasome activation after RNA virus infection. Proc. Natl Acad. Sci. USA..

[12] Tur J (2020). Mitofusin 2 in macrophages links mitochondrial ROS production, cytokine release, phagocytosis, autophagy, and bactericidal activity. Cell Rep..

[13] Paik S, Jo EK (2020). An interplay between autophagy and immunometabolism for host defense against mycobacterial infection. Front Immunol..

[14] Kim H, Lee JY, Park KJ, Kim WH, Roh GS (2016). A mitochondrial division inhibitor, Mdivi-1, inhibits mitochondrial fragmentation and attenuates kainic acid-induced hippocampal cell death. BMC Neurosci..

[15] Luo X (2019). Mitochondrial division inhibitor 1 attenuates mitophagy in a rat model of acute lung injury. Biomed. Res. Int..

[16] Jha AK (2015). Network integration of parallel metabolic and transcriptional data reveals metabolic modules that regulate macrophage polarization. Immunity.

[17] O’Neill LA, Pearce EJ (2016). Immunometabolism governs dendritic cell and macrophage function. J. Exp. Med..

[18] Langston PK, Shibata M, Horng T (2017). Metabolism supports macrophage activation. Front Immunol..

[19] Son MJ (2015). Mitofusins deficiency elicits mitochondrial metabolic reprogramming to pluripotency. Cell Death Differ..

[20] TeSlaa T, Teitell MA (2014). Techniques to monitor glycolysis. Methods Enzymol..

[21] Yuk JM (2015). Orphan nuclear receptor ERRalpha controls macrophage metabolic signaling and A20 expression to negatively regulate TLR-induced inflammation. Immunity.

[22] Scialo, F. et al. Mitochondrial complex I derived ROS regulate stress adaptation in Drosophila melanogaster. *Redox Biol*. **32**, 101450 (2020). 10.1016/j.redox.2020.101450 PMC7264463 32146156

[23] Murphy MP (2009). How mitochondria produce reactive oxygen species. Biochem J..

[24] Sun W (2009). Mitochondrial mutations contribute to HIF1alpha accumulation via increased reactive oxygen species and up-regulated pyruvate dehydrogenease kinase 2 in head and neck squamous cell carcinoma. Clin. Cancer Res..

[25] Chacko BK (2011). Mitochondria-targeted ubiquinone (MitoQ) decreases ethanol-dependent micro and macro hepatosteatosis. Hepatology.

[26] Zhang Y (2019). Listeria hijacks host mitophagy through a novel mitophagy receptor to evade killing. Nat. Immunol..

[27] Demishtein-Zohary K, Azem A (2017). The TIM23 mitochondrial protein import complex: function and dysfunction. Cell Tissue Res..

[28] Zhao T (2012). Central role of mitofusin 2 in autophagosome-lysosome fusion in cardiomyocytes. J. Biol. Chem..

[29] Kim TS (2017). Ohmyungsamycins promote antimicrobial responses through autophagy activation via AMP-activated protein kinase pathway. Sci. Rep..

[30] Hansen TE, Johansen T (2011). Following autophagy step by step. BMC Biol..

[31] Seok JY (2018). Upregulation of AMPK by 4-O-methylascochlorin promotes autophagy via the HIF-1alpha expression. J. Cell Mol. Med..

[32] Xie Y (2018). Ischemic preconditioning attenuates ischemia/reperfusion-induced kidney injury by activating autophagy via the SGK1 signaling pathway. Cell Death Dis..

[33] Lai HH (2018). HIF-1alpha promotes autophagic proteolysis of Dicer and enhances tumor metastasis. J. Clin. Invest..

[34] de Brito OM, Scorrano L (2008). Mitofusin 2 tethers endoplasmic reticulum to mitochondria. Nature.

[35] Parekh A (2009). Calcium signalling: mitofusins promote interorganellar crosstalk. Curr. Biol..

[36] Silva Ramos E, Larsson NG, Mourier A (2016). Bioenergetic roles of mitochondrial fusion. Biochim Biophys. Acta.

[37] Yasukawa K (2009). Mitofusin 2 inhibits mitochondrial antiviral signaling. Sci. Signal.

[38] Koshiba T, Yasukawa K, Yanagi Y, Kawabata S (2011). Mitochondrial membrane potential is required for MAVS-mediated antiviral signaling. Sci. Signal.

[39] Luo Z (2019). Epigoitrin, an alkaloid from isatis indigotica, reduces H1N1 infection in stress-induced susceptible model in vivo and in vitro. Front Pharm..

[40] Correa da Silva F (2019). Ghrelin effects on mitochondrial fitness modulates macrophage function. Free Radic. Biol. Med.

[41] Corcoran SE, O’Neill LA (2016). HIF1alpha and metabolic reprogramming in inflammation. J. Clin. Invest..

[42] Prigione A (2014). HIF1alpha modulates cell fate reprogramming through early glycolytic shift and upregulation of PDK1-3 and PKM2. Stem Cells.

[43] Hyttinen JM, Niittykoski M, Salminen A, Kaarniranta K (2013). Maturation of autophagosomes and endosomes: a key role for Rab7. Biochim Biophys. Acta.

[44] Hutagalung AH, Novick PJ (2011). Role of Rab GTPases in membrane traffic and cell physiology. Physiol. Rev..

[45] Jager S (2004). Role for Rab7 in maturation of late autophagic vacuoles. J. Cell Sci..

[46] Wong YC, Kim S, Peng W, Krainc D (2019). Regulation and function of mitochondria-lysosome membrane contact sites in cellular homeostasis. Trends Cell Biol..

[47] Wong YC, Ysselstein D, Krainc D (2018). Mitochondria-lysosome contacts regulate mitochondrial fission via RAB7 GTP hydrolysis. Nature.

[48] Zhang, X., Goncalves, R. & Mosser, D. M. The isolation and characterization of murine macrophages. *Curr. Protoc. Immunol.* **Chapter 14**, Unit 14.1 (2008). 10.1002/0471142735.im1401s83 PMC2834554 19016445

[49] Köster J, Rahmann S (2012). Snakemake—a scalable bioinformatics workflow engine. Bioinformatics.

[50] Andrews, S. *FastQC: A Quality Control Tool for High Throughput Sequence Data*. Babraham Bioinformatics, Babraham Institute (2019).

[51] Martin, M. Cutadapt removes adapter sequences from high-throughput sequencing reads. *EMBnet.journal*, **17**, 3 (2011).

[52] Patro R, Duggal G, Love MI, Irizarry RA, Kingsford C (2017). Salmon provides fast and bias-aware quantification of transcript expression. Nat. Methods.

[53] Soneson, C., Love, M. I. & Robinson, M. D. Differential analyses for RNA-seq: transcript-level estimates improve gene-level inferences. *F1000Res.* **4**, 1521 (2015). 10.12688/f1000research.7563.1 PMC4712774 26925227

[54] Love MI, Huber W, Anders S (2014). Moderated estimation of fold change and dispersion for RNA-seq data with DESeq2. Genome Biol..

[55] Dagda RK (2009). Loss of PINK1 function promotes mitophagy through effects on oxidative stress and mitochondrial fission. J. Biol. Chem..

[56] Kim JK (2017). MIR144* inhibits antimicrobial responses against *Mycobacterium tuberculosis* in human monocytes and macrophages by targeting the autophagy protein DRAM2. Autophagy.

