# Supplementary material for: Mitofusin-2 boosts innate immunity through the maintenance of aerobic glycolysis and activation of xenophagy in mice
Source: Commun Biol. 2021 May 10;4:548. doi: 10.1038/s42003-021-02073-6 (PMC8110749; doi:10.1038/s42003-021-02073-6)
Supplement: Supplementary file 1 — Supplementary Information [file 42003_2021_2073_MOESM1_ESM.pdf]

Supplementary Figure 1

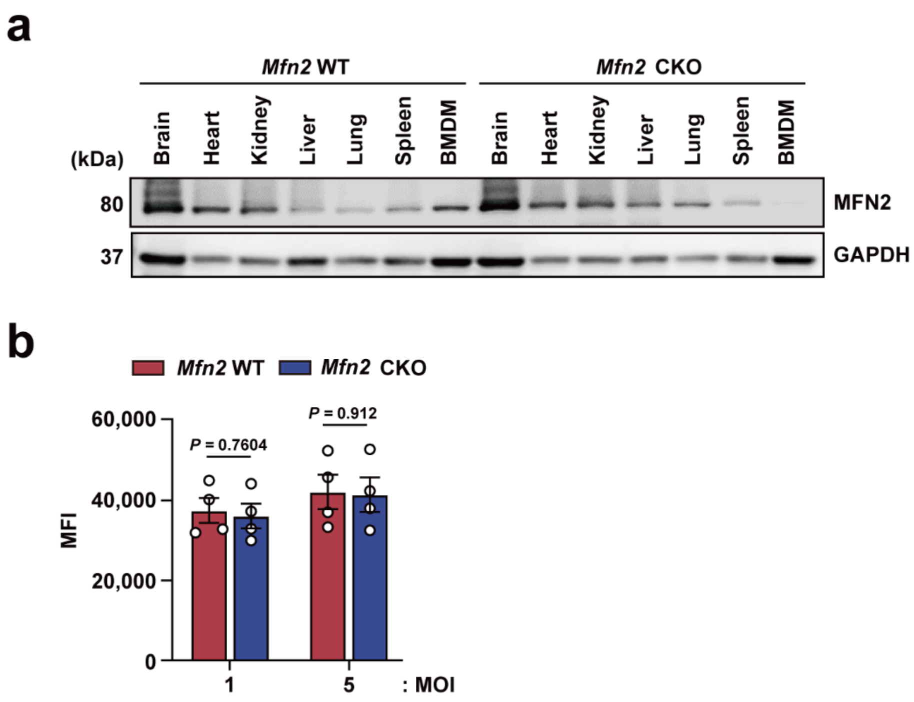

Supplementary figure 1. **Confirmation of MFN2 knockout in myeloid cell and comparative analysis of phagocytosis between *Mfn2* WT and *Mfn2* CKO BMDMs. a,** Western blot analysis to detect the level of MFN2 in tissue samples and BMDMs from *Mfn2* WT or *Mfn2* CKO mice. GAPDH was used as loading control. **b,** Phagocytosis measured by flow cytometric analysis in *Mfn2* WT and *Mfn2* CKO BMDMs infected with Mtb-ERFP at the indicated MOIs for 4 h (n=4). Data are presented as mean±SEM. Two-tailed Student's *t* test is used to calculate the significance (**b**). MFI, mean fluorescence intensities.

Supplementary Figure 2

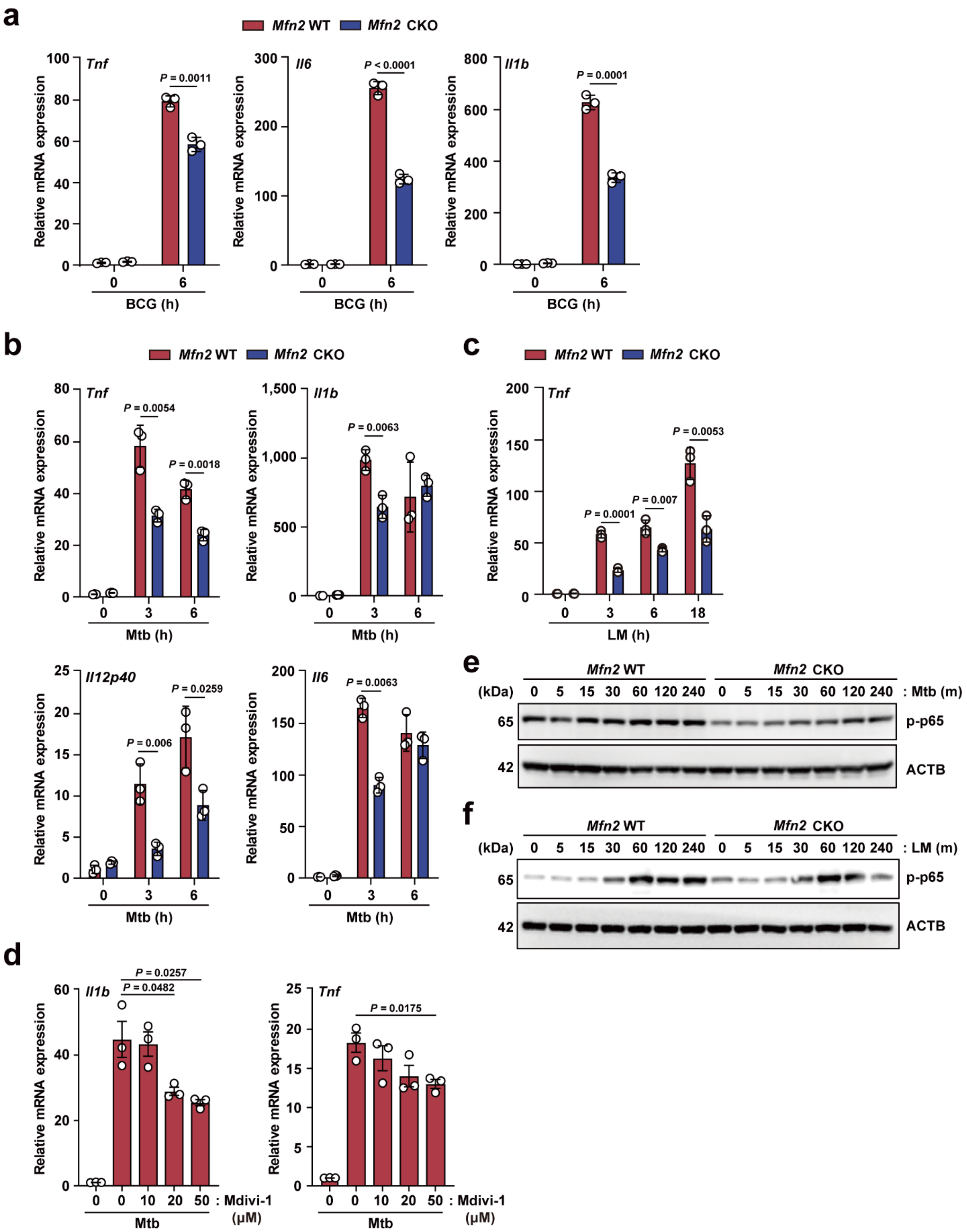

Supplementary figure 2. MFN2 is required for inflammatory responses in macrophages following bacterial infection. a, qPCR analysis of *Tnf*, *Il6,* and *Il1b* in BCG (MOI 5 for indicated time)-infected *Mfn2* WT or *Mfn2* CKO BMDMs (n=3). **b,** qPCR analysis of *Tnf, Il1b, Il12p40* and *Il6* in *Mfn2* WT or *Mfn2* CKO PMs infected with Mtb (MOI 5) for indicated time (n=3). **c,** qPCR analysis of *Tnf* in *Mfn2* WT and *Mfn2* CKO PMs infected with LM (MOI 5) for indicated time (n=3). **d,** BMDMs from *Mfn2* WT were infected with Mtb (MOI 5) and treated with indicated dose of Mdivi-1 and analyzed for mRNA expression of *Il1b* and *Tnf* using qPCR. **e,f,** Western blot analysis of phospho-p65 (NF-κB) and ACTB in Mtb (MOI 5) infected PMs (**e**) or LM (MOI 5) infected BMDMs (**f**). Data presented as mean±SD (**a-c**) or mean±SEM (**d**) are representative of 2-3 independent experiments (**a-f**). Two-tailed Student's *t* test is used to calculate the significance (**a-c,d**).

Supplementary Figure 3

a

Class	Metabolites	*Mfn2* WT Un	*Mfn2* WT Mtb	*Mfn2* CKO Un	*Mfn2* CKO Mtb
Organic acids	Lactic acid	1.14	1.07	0.97	0.82
	Pyruvic acid	0.75	1.01	1.38	0.87
	Urea	0.29	1.13	1.10	1.47
	Succinic acid	0.56	1.36	0.59	1.48
	Itaconic acid	0.01	2.05	0.01	1.93
	Citric acid	0.64	1.07	1.32	0.98
Amino acids	Isoleucine	0.24	0.56	1.05	2.15
	Glycine	0.60	1.40	0.88	1.11
	Serine	0.02	0.48	0.76	2.73
	Threonine	0.13	0.68	1.15	2.03
	5-oxo-proline	0.76	1.11	0.93	1.21
Sugar & sugar alcohols	Saccharide 1	0.42	0.77	1.24	1.56
	myo-Inositol	1.92	0.80	0.73	0.55
	Saccharide 2	0.00	1.96	0.00	2.03
Etc.	Methylphosphate	1.96	1.00	0.53	0.50
	4-Hydroxybutyric acid	0.65	0.86	1.24	1.25
	Phosphoethanolamine	0.33	1.62	0.76	1.29
N.I	Non-identifications 1	0.02	0.04	1.99	1.96
	Non-identifications 2	0.69	1.04	1.23	1.03
	Non-identifications 3	0.69	0.84	1.50	0.97
	Non-identifications 4	1.18	1.27	0.77	0.79
	Non-identifications 5	0.00	2.47	0.00	1.53
	Non-identifications 6	0.46	0.77	1.38	1.39

Color scale: 0 – 1 – 2

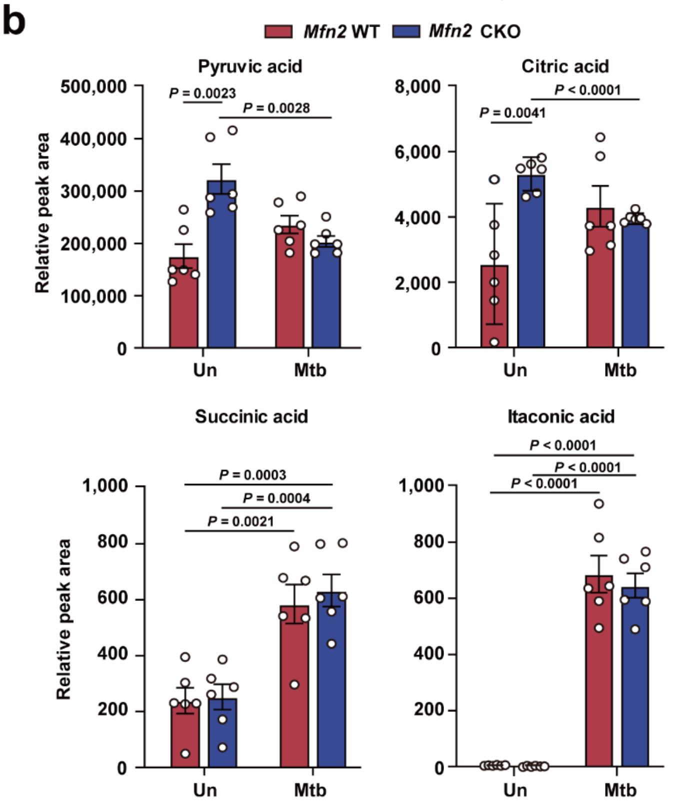

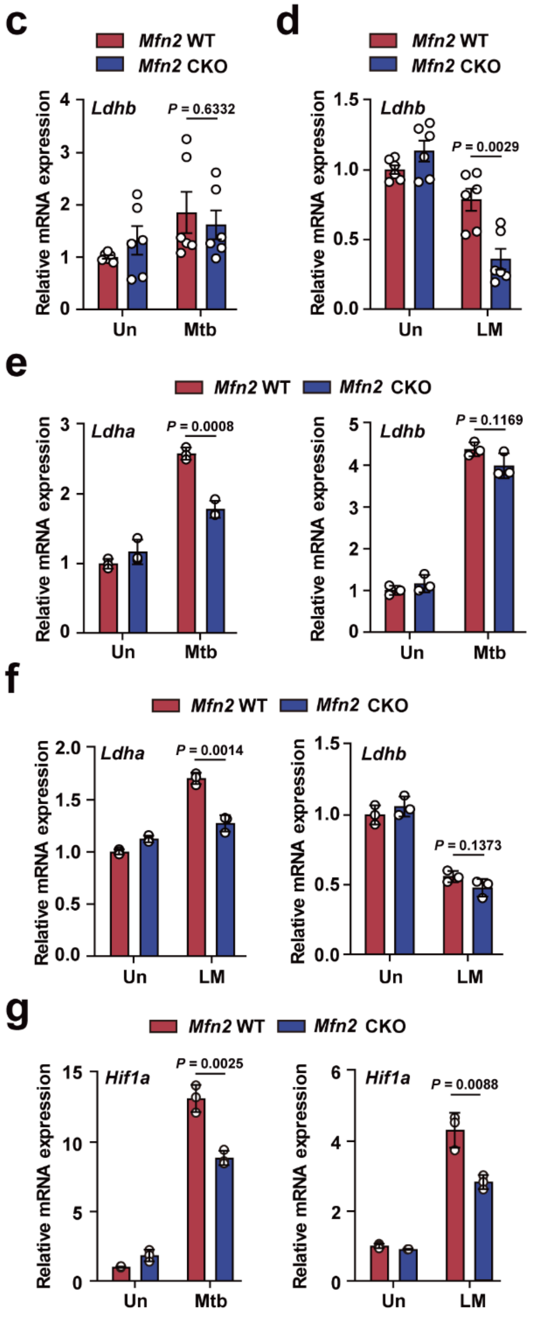

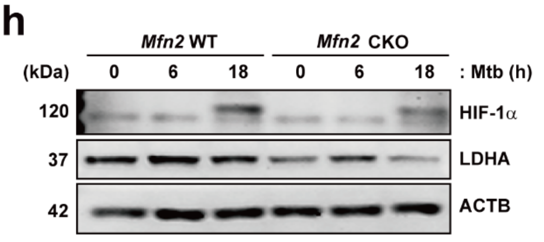

Supplementary figure 3. Comparative analysis of metabolite profiles and *Ldha* and *Hif1a* mRNA expression in macrophages following Mtb infection. a, Heat map representation of the relative concentration of significantly discriminant metabolites (VIP > 0.7) based on the PLS-DA model. **b,** The bar plots showed relative concentration of identified organic acids that was calculated by relative peak area from GC-TOF/MS analysis. **c,d,** qPCR analysis of *Ldhb* in *Mfn2* WT or *Mfn2* CKO BMDMs infected with Mtb (MOI 5) (**c**) and LM (MOI 5) (**d**) for indicated time (n=6). **e-g,** qPCR analysis of *Ldha*, *Ldhb* and *Hif1a* in PMs infected with Mtb (MOI 5) (**e,g**) and LM (MOI 5) (**f,g**) for indicated time (n=3). **h,** Western blot analysis of HIF-1α, LDHA and ACTB in Mtb (MOI 5) infected BMDMs. Data are presented as mean±SEM (**c,d**) or mean±SD (**e-g**); pooled from three independent experiments (**c,d**) or representative of 2-3 independent experiments (**e-h**). The data were analyzed by the multiple t-test with Holm-Sidak correction (**a,b**) or two-tailed Student's *t* test is used to calculate the significance (**c-g**). Un, uninfected.

Supplementary Figure 4

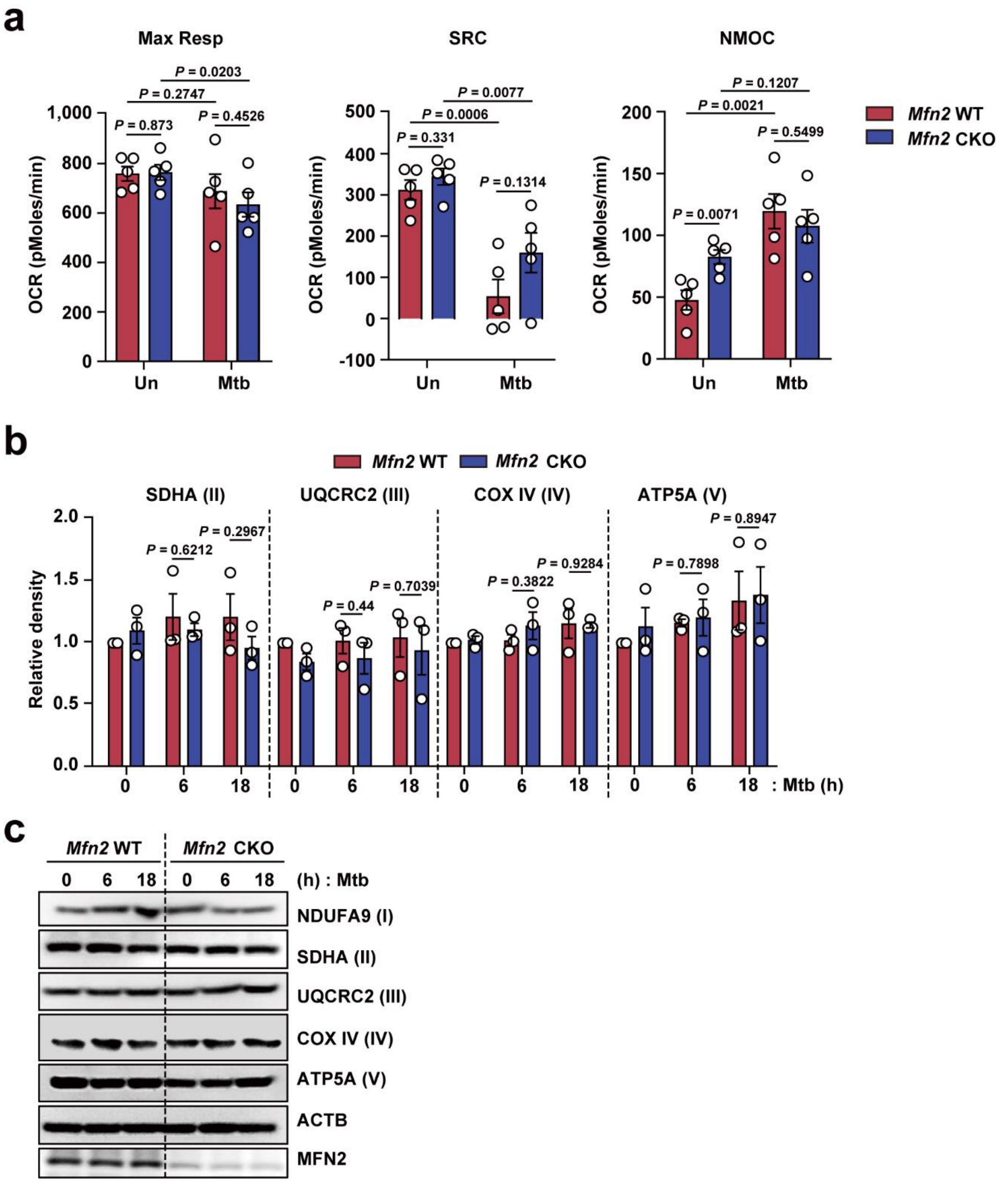

Supplementary figure 4. Comparative analysis of OXPHOS protein levels between *Mfn2* WT and *Mfn2* CKO macrophages following Mtb infection. a, Maximal respiration (Max Resp), spare respiratory capacity (SRC) and non-mitochondrial oxidative capacity (NMOC) parameters

from the OCR results in Fig. **4a** are shown. **b,** Densitometry analysis of OXPHOS proteins (II to V) for blots shown in Fig. **4d**. Two-tailed Student's *t* test is used to calculate the significance (n=3). **c,** Western blot analysis showing the protein levels of OXPHOS complexes in *Mfn2* WT and *Mfn2* CKO PMs after Mtb (MOI 5) infection for indicated time. Representative of 2-3 independent experiments are shown. Two-tailed Student's *t* test is used to calculate the significance (**a,b**). Un, uninfected.

Supplementary Figure 5

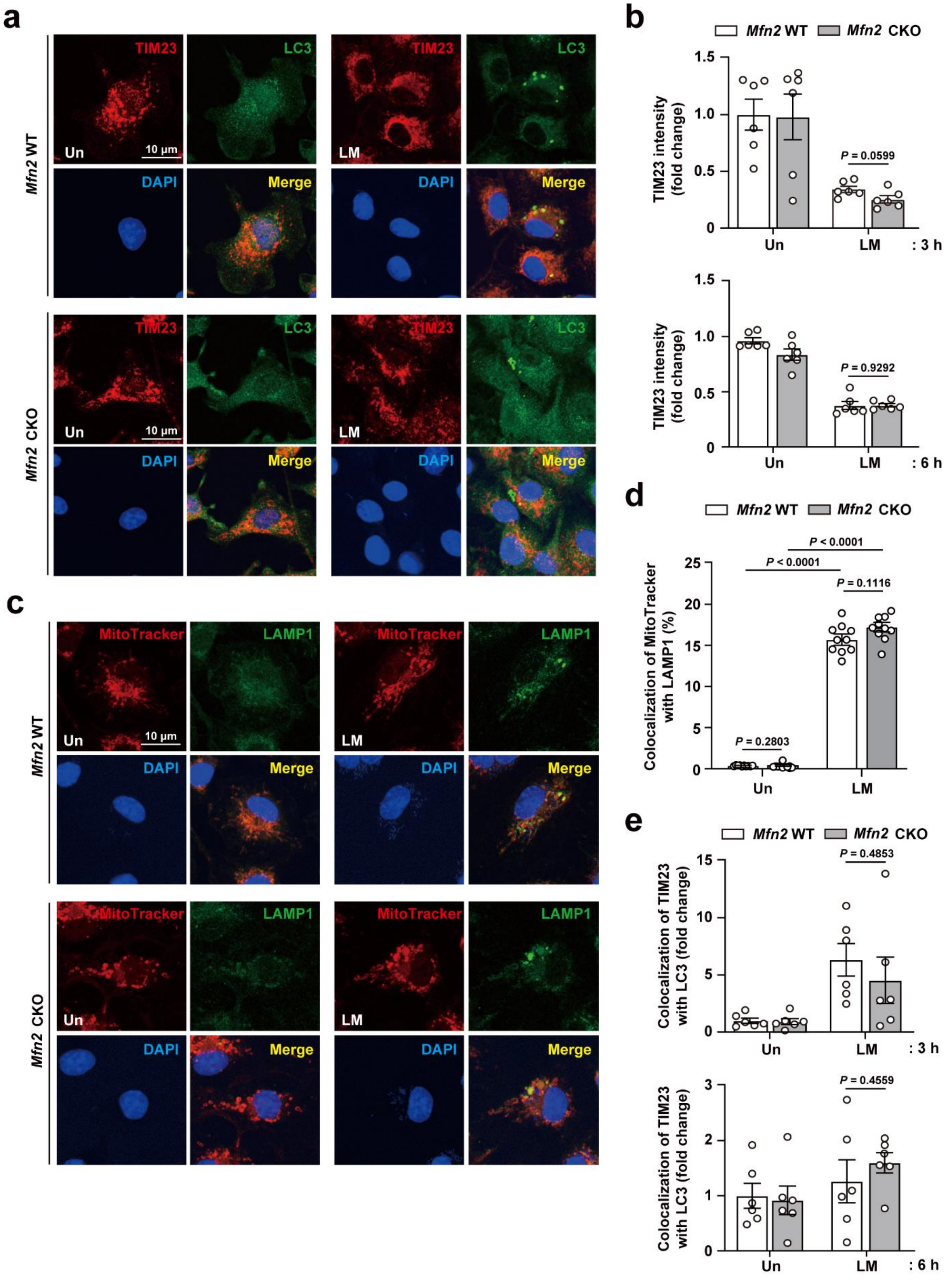

Supplementary figure 5. Mitophagy analysis between *Mfn2* WT and *Mfn2* CKO macrophages during LM infection. a,b,e, BMDMs from *Mfn2* WT and *Mfn2* CKO were infected with LM (MOI 5) for 3 or 6 h. Cells were stained with TIM23 (red), LC3 (green), and DAPI (for nuclei; blue). **a,** Representative images for LC3 (green) colocalization with TIM23 (red) in *Mfn2* WT and *Mfn2* CKO BMDMs after LM infection for 3 h. Scale bars, 10 μm. **b,** Quantitative data of TIM23 intensity analysis. 50 cells in 6 fields were counted in each group from two different experiments. **e,** Quantitative data of colocalization of LC3 and TIM23. 50 cells in 6 fields were counted in each group from two different experiments. **c,d,** BMDMs from *Mfn2* WT and *Mfn2* CKO were infected with LM (MOI 5) for 3 h. Cells were stained with MitoTracker (red), LAMP1 (green), and DAPI (for nuclei; blue). **c,** Representative images. **d,** Quantitative data of colocalization of Mitotracker and LAMP1. 50 cells in 10 fields were counted in each group from two different experiments. Scale bars, 10 μm. Data are presented as mean±SEM (**b,d,e**) and are representative of two independent experiments. Two-tailed Student's *t* test is used to calculate the significance (**b,d,e**). Un, uninfected.

Supplementary Figure 6

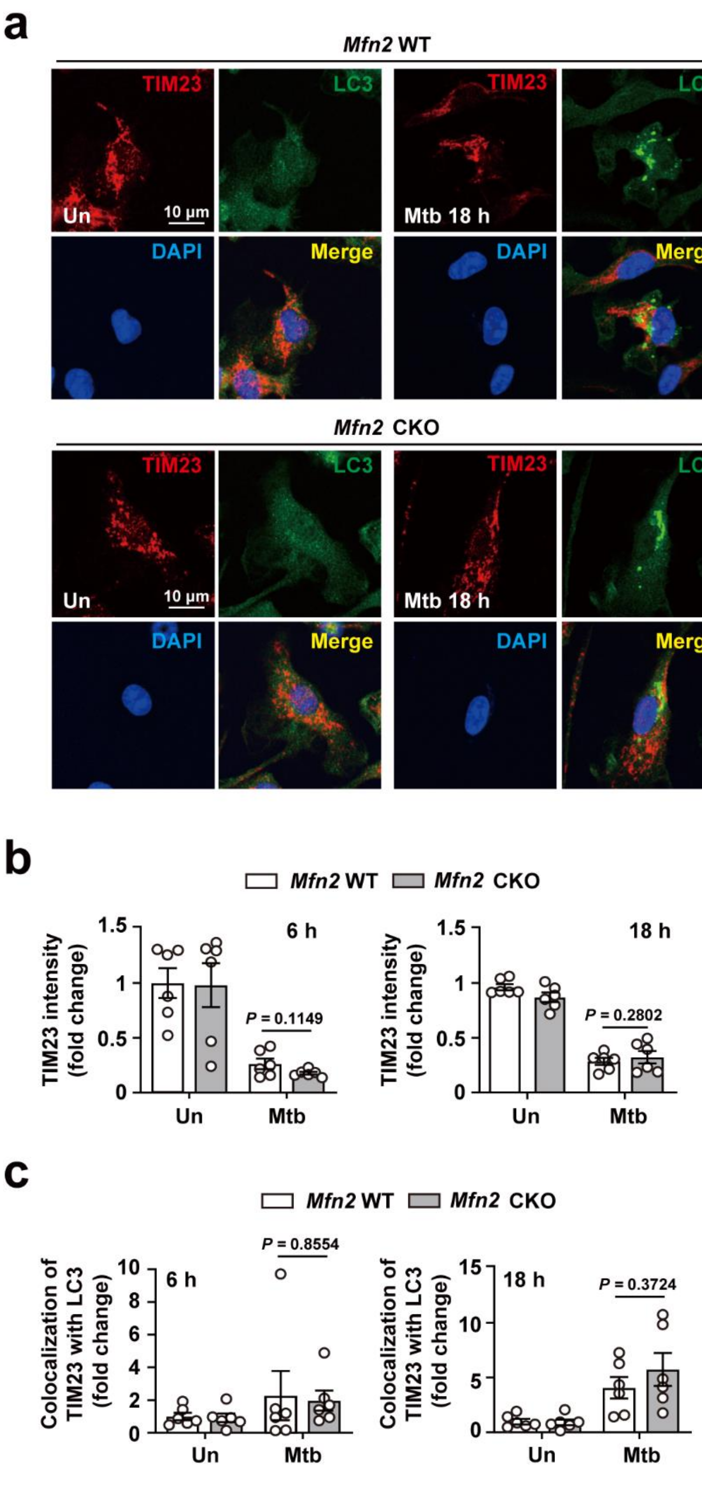

Supplementary figure 6. Mitophagy analysis between *Mfn2* WT and *Mfn2* CKO macrophages during Mtb infection. **a-c,** Mtb (MOI 5)-infected BMDMs from *Mfn2* WT and *Mfn2*

CKO mice were stained with TIM23 (red), LC3 (green), and DAPI (for nuclei; blue). **a,** Representative images for LC3 colocalization with TIM23; Scale bars, 10 μm. **b,** Quantitative data of TIM23 intensity analysis. 50 cells in 6 fields were counted in each group from two different experiments. **c,** Quantitative data of colocalization of LC3 and TIM23. 50 cells in 6 fields were counted in each group from two different experiments. Two-tailed Student's *t* test is used to calculate the significance (**b,c**). Data are presented as mean±SEM and are representative of three independent experiments. Un, uninfected.

Supplementary Figure 7

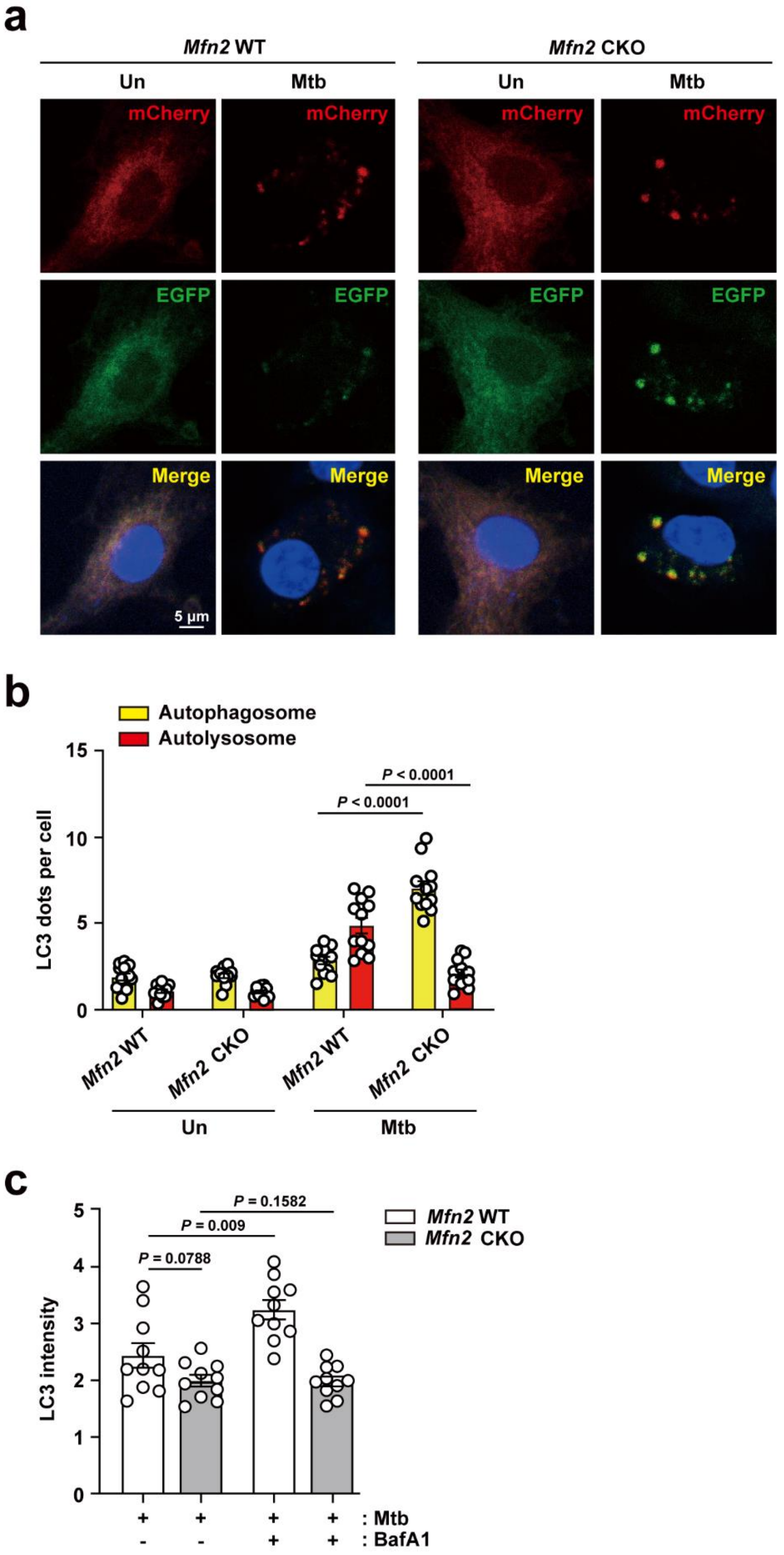

Supplementary figure 7. MFN2 is involved in the autophagic flux in macrophages during Mtb infection. a,b, *Mfn2* WT and *Mfn2* CKO BMDMs were transduced with retrovirus expressing a tandem LC3B plasmid (mCherry-EGFP-LC3B). Cells were infected with Mtb (MOI 5) for 6 h and analyzed using confocal microscopy. **a,** Representative images. Scale bars, 5 μm. **b,** The number of yellow and red LC3 dots per cell was quantified. **c,** BMDMs from *Mfn2* WT and *Mfn2* CKO were pretreated with bafilomycin A1 (BafA1, 200 nM for 2 h) and infected with Mtb-ERFP (MOI 5). Cells were stained with LC3 and intensity was analyzed using confocal microscopy. Quantitative data of LC3 intensity is shown. 50 cells in 10 fields were counted in each group from two different experiments. Data are presented as mean±SEM (**b,c**) and are representative of two independent experiments. Two-tailed Student's *t* test is used to calculate the significance (**b,c**). Un, uninfected.

Supplementary Figure 8

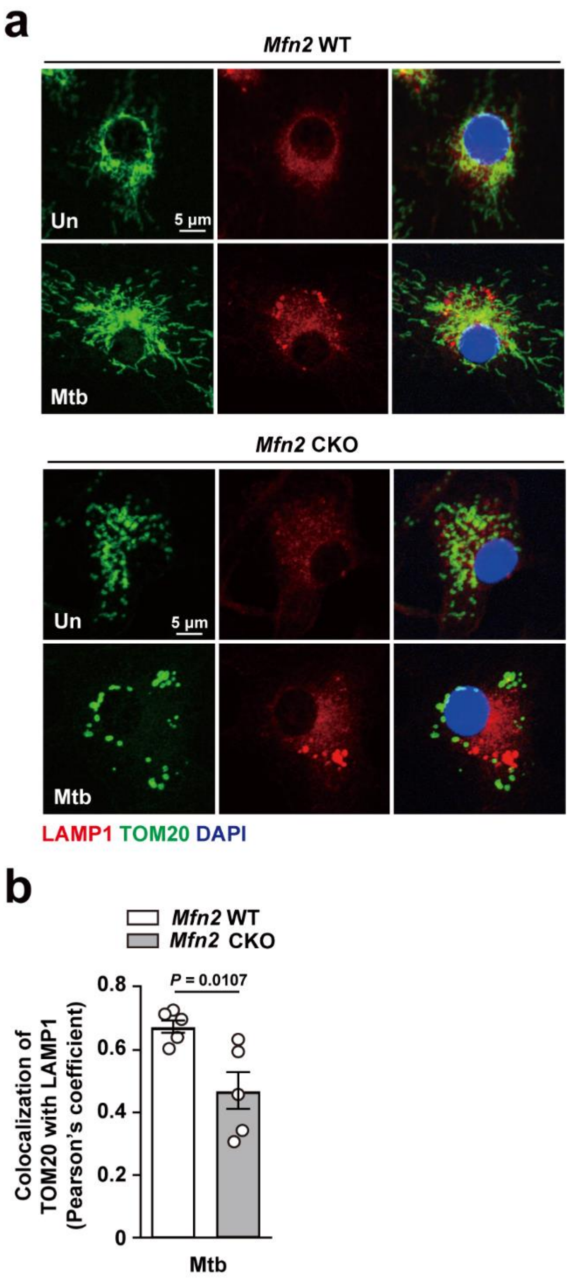

Supplementary figure 8. Reduction in colocalization of TOM20 with LAMP1 in *Mfn2* CKO macrophages after bacterial infection. a,b, *Mfn2* WT or *Mfn2* CKO BMDMs infected with Mtb (MOI 5, for 6 h) were stained with LAMP1 (red), TOM20 (green) and DAPI (blue). **a,** Representative confocal images are shown. Scale bars, 5 μm. **b,** Quantitative analysis of

Pearson's colocalization coefficient between TOM20 and LAMP1. 50 cells in 5 fields were counted in each group from two different experiments. Data are presented as mean±SEM and are representative of two independent experiments. Two-tailed Student's *t* test is used to calculate the significance (**b**). Un, uninfected.

Supplementary Figure 9

Fig. 2f

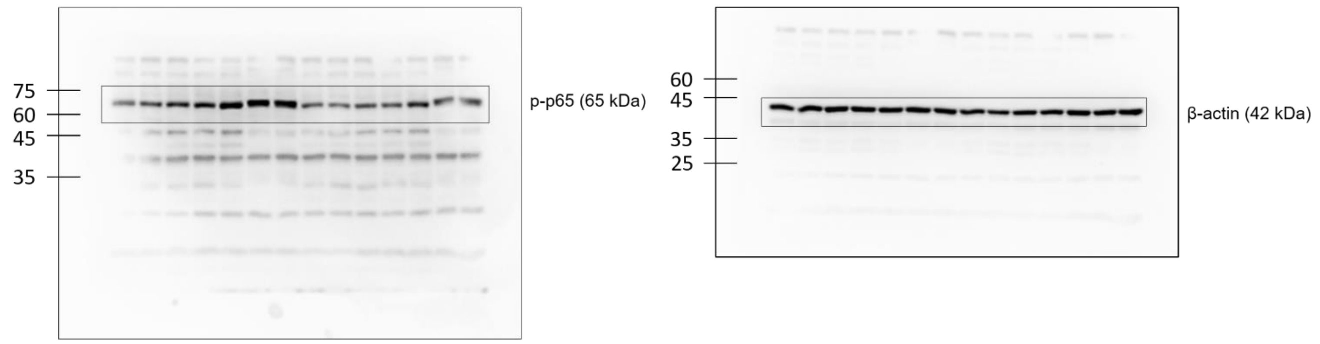

Fig. 4d

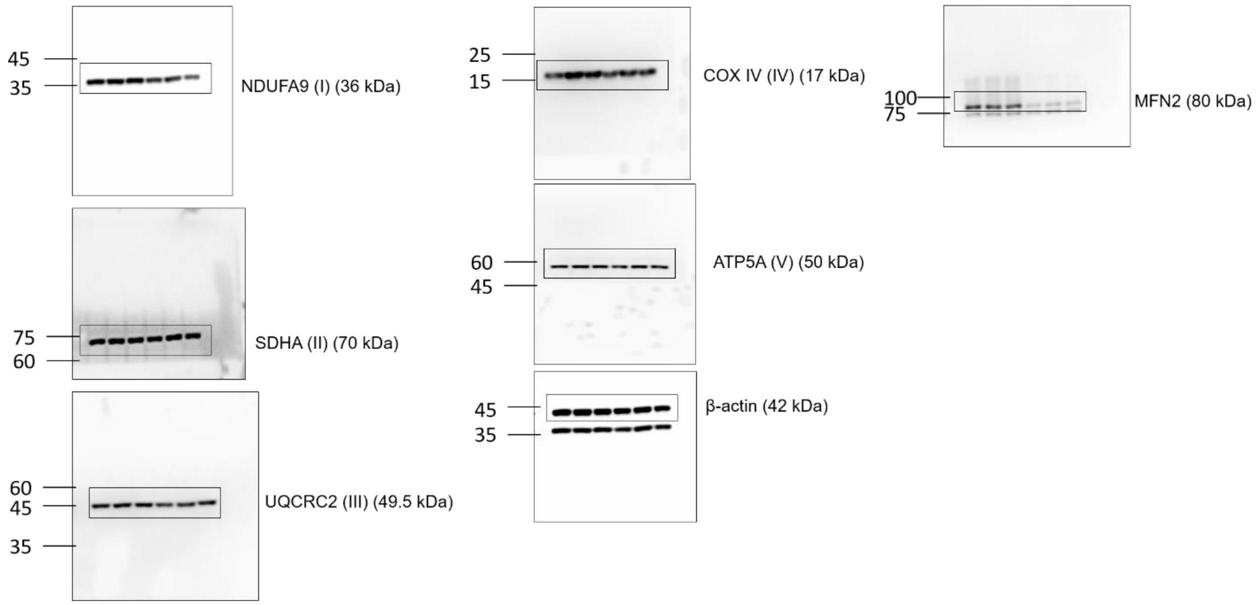

Fig. 5d

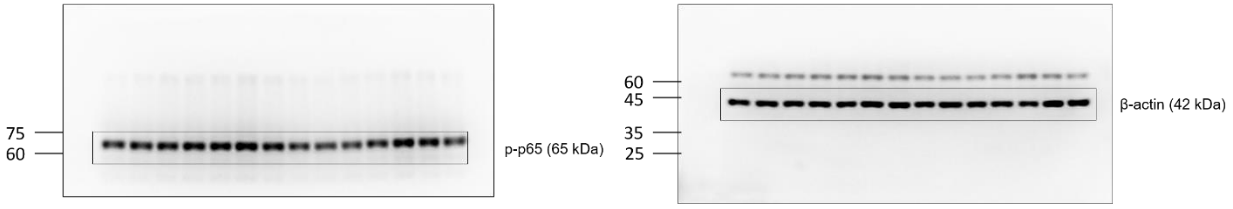

Fig. 8a

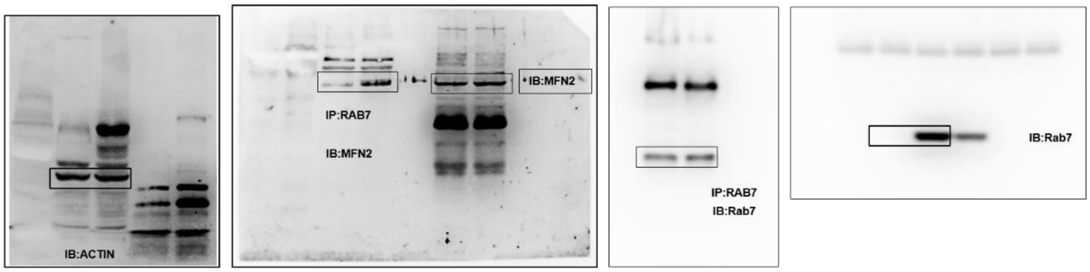

Supplementary Fig. 1a

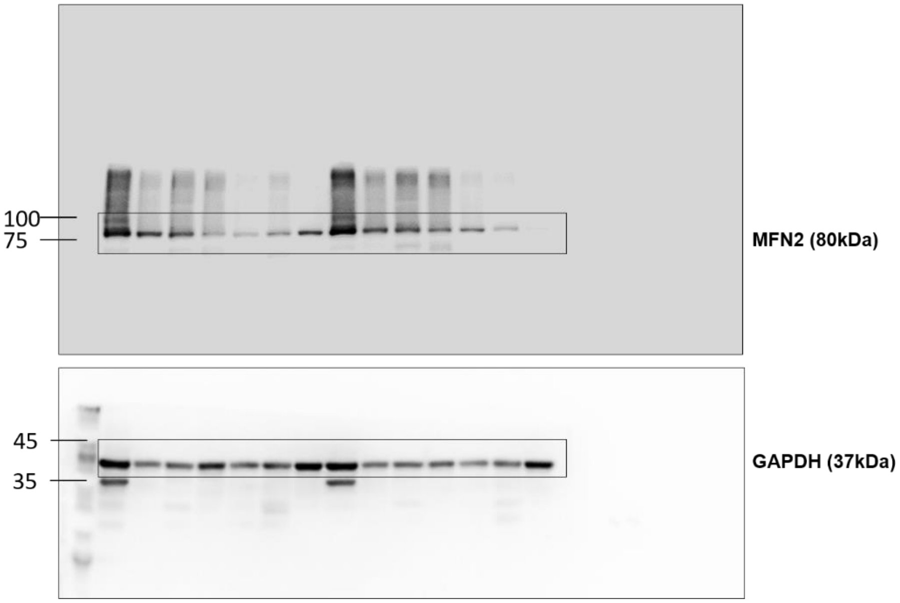

Supplementary Fig. 2d

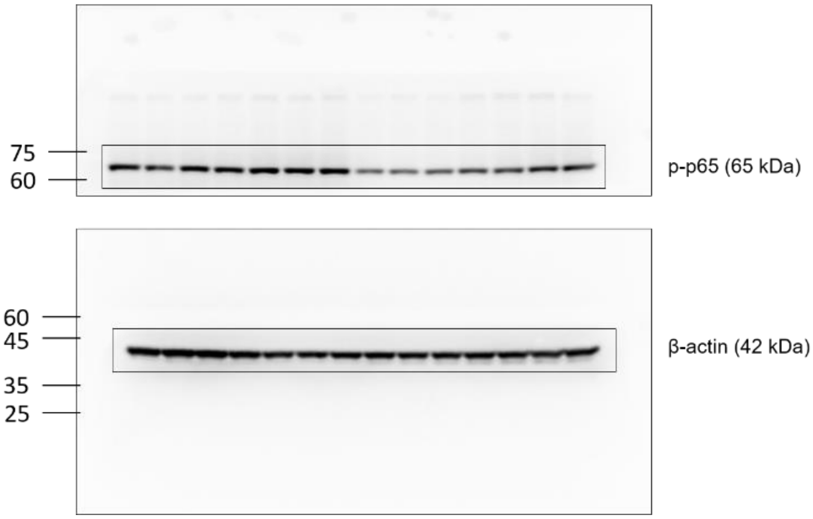

Supplementary Fig. 2e

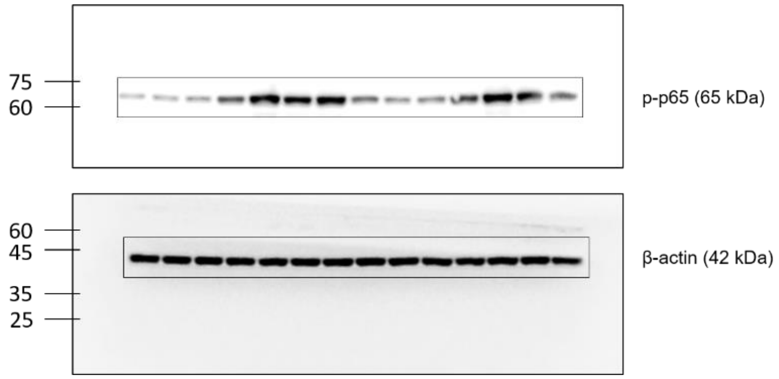

Supplementary Fig. 3h

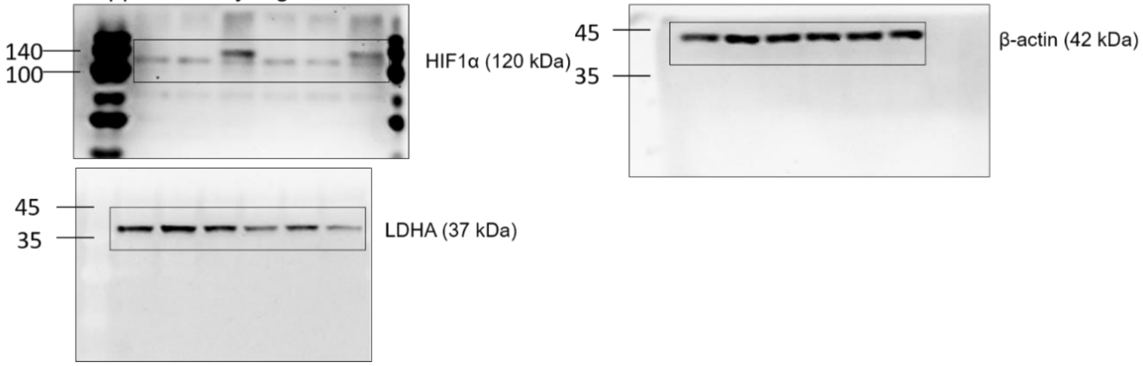

Supplementary Fig. 4c

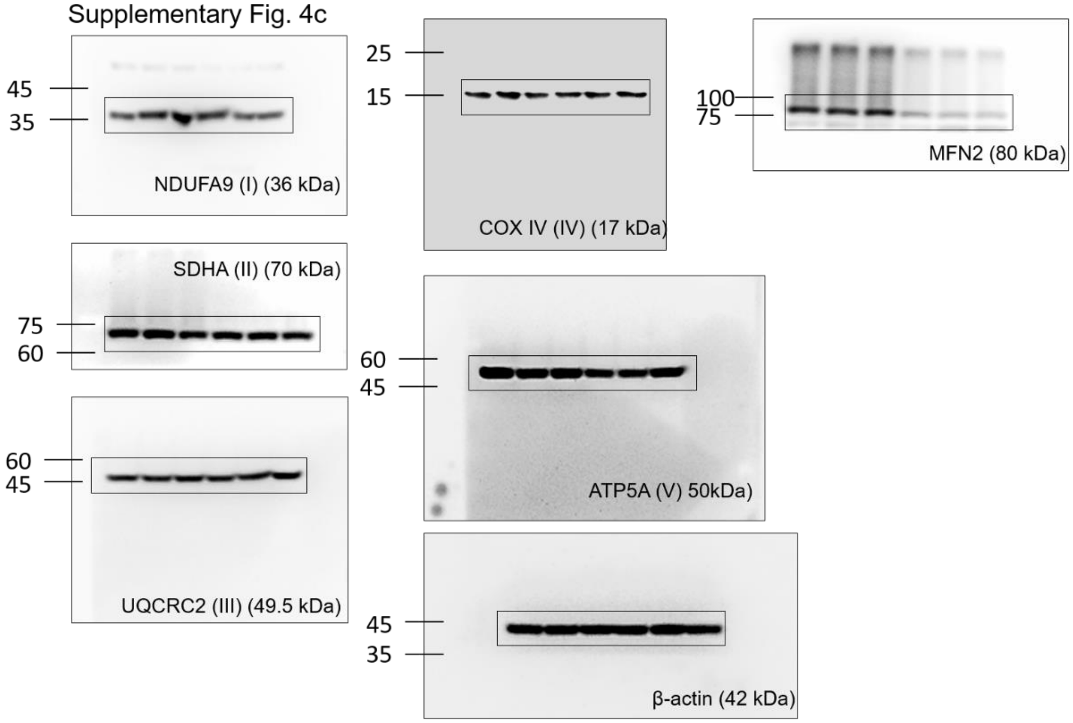

Supplementary figure 9. Uncropped images of western blot. Black boxes show the region

cropped from each blot and presented. Each figure corresponds to the western blots in the indicated figure number.
