# Supplementary material for: Mitofusin-2 boosts innate immunity through the maintenance of aerobic glycolysis and activation of xenophagy in mice
Source: Commun Biol. 2021 May 10;4:548. doi: 10.1038/s42003-021-02073-6 (PMC8110749; doi:10.1038/s42003-021-02073-6)
Supplement: Supplementary file 2 — Descriptions of Additional Supplementary Files [file 42003_2021_2073_MOESM2_ESM.pdf]

Description of Additional Supplementary Files

Supplementary Data 1
Description: The source data underlying the graphs and charts presented in the main figures.
