# Supplementary material for: Mitofusin-2 boosts innate immunity through the maintenance of aerobic glycolysis and activation of xenophagy in mice
Source: Commun Biol. 2021 May 10;4:548. doi: 10.1038/s42003-021-02073-6 (PMC8110749; doi:10.1038/s42003-021-02073-6)
Supplement: Supplementary file 4 — Reporting Summary [file 42003_2021_2073_MOESM4_ESM.pdf]

nature research

Corresponding author(s): Eun-Kyeong Jo

Last updated by author(s): Mar 19, 2021

Reporting Summary

Nature Research wishes to improve the reproducibility of the work that we publish. This form provides structure for consistency and transparency in reporting. For further information on Nature Research policies, see our Editorial Policies and the Editorial Policy Checklist.

Statistics

For all statistical analyses, confirm that the following items are present in the figure legend, table legend, main text, or Methods section.

n/a	Confirmed	
☐	☒	The exact sample size (n) for each experimental group/condition, given as a discrete number and unit of measurement
☐	☒	A statement on whether measurements were taken from distinct samples or whether the same sample was measured repeatedly
☐	☒	The statistical test(s) used AND whether they are one- or two-sided<br>*Only common tests should be described solely by name; describe more complex techniques in the Methods section.*
☒	☐	A description of all covariates tested
☐	☒	A description of any assumptions or corrections, such as tests of normality and adjustment for multiple comparisons
☐	☒	A full description of the statistical parameters including central tendency (e.g. means) or other basic estimates (e.g. regression coefficient) AND variation (e.g. standard deviation) or associated estimates of uncertainty (e.g. confidence intervals)
☐	☒	For null hypothesis testing, the test statistic (e.g. F, t, r) with confidence intervals, effect sizes, degrees of freedom and P value noted<br>*Give P values as exact values whenever suitable.*
☒	☐	For Bayesian analysis, information on the choice of priors and Markov chain Monte Carlo settings
☒	☐	For hierarchical and complex designs, identification of the appropriate level for tests and full reporting of outcomes
☒	☐	Estimates of effect sizes (e.g. Cohen's d, Pearson's r), indicating how they were calculated

Our web collection on statistics for biologists contains articles on many of the points above.

Software and code

Policy information about availability of computer code

Data collection	We have provided the description of all software used in the study in detail.
Data analysis	We have provided the description of all software used in the study in detail.

For manuscripts utilizing custom algorithms or software that are central to the research but not yet described in published literature, software must be made available to editors and reviewers. We strongly encourage code deposition in a community repository (e.g. GitHub). See the Nature Research guidelines for submitting code & software for further information.

Data

Policy information about availability of data

All manuscripts must include a data availability statement. This statement should provide the following information, where applicable:

- Accession codes, unique identifiers, or web links for publicly available datasets
- A list of figures that have associated raw data
- A description of any restrictions on data availability

Data that support the findings of this study are available from the corresponding author upon reasonable request.

Field-specific reporting

Please select the one below that is the best fit for your research. If you are not sure, read the appropriate sections before making your selection.

☒ Life sciences ☐ Behavioural & social sciences ☐ Ecological, evolutionary & environmental sciences

For a reference copy of the document with all sections, see nature.com/documents/nr-reporting-summary-flat.pdf

Life sciences study design

All studies must disclose on these points even when the disclosure is negative.

Sample size	Sample numbers were predetermined based on pilot studies and sample sizes were similar to generally employed in the field.
Data exclusions	No data were excluded from the analysis.
Replication	We confirm that all attempts at replication were successful.
Randomization	The samples/animals were selected randomly. In each group, gender/age was matched between control and experimental group.
Blinding	We did not perform blinding test. The samples/animals were selected randomly and they were gender/age matched.

Reporting for specific materials, systems and methods

We require information from authors about some types of materials, experimental systems and methods used in many studies. Here, indicate whether each material, system or method listed is relevant to your study. If you are not sure if a list item applies to your research, read the appropriate section before selecting a response.

Materials & experimental systems

n/a	Involved in the study	
☐	☒	Antibodies
☐	☒	Eukaryotic cell lines
☒	☐	Palaeontology and archaeology
☐	☒	Animals and other organisms
☒	☐	Human research participants
☒	☐	Clinical data
☒	☐	Dual use research of concern

Methods

n/a	Involved in the study	
☒	☐	ChIP-seq
☐	☒	Flow cytometry
☒	☐	MRI-based neuroimaging

Antibodies

Antibodies used	We have provided the description of all antibodies used in the study in detail.
Validation	Each primary antibody used in this study has its own validation for its species and application, which was stated on the manufacturer's website.

Eukaryotic cell lines

Policy information about cell lines

Cell line source(s)	We obtained RAW264.7 (TIB-71) cell line from American Type Culture Collection (ATCC, Manassas, VA).
Authentication	RAW264.7 (TIB-71) cell line obtained from American Type Culture Collection (ATCC, Manassas, VA) have been authenticated by provider.
Mycoplasma contamination	Cell lines were routinely tested for mycoplasma using a commercially available kit (MycoAlert, Lonza).
Commonly misidentified lines (See ICLAC register)	N.A.

Animals and other organisms

Policy information about studies involving animals; ARRIVE guidelines recommended for reporting animal research

Laboratory animals	Mice wild type C57BL/6, Mfn2flox/flox, Mfn2flox/flox;LysM-Cre+, sex matched, aged 6-8 weeks
Wild animals	This study did not involve wild animals.
Field-collected samples	This study did not involve field-collected samples.
Ethics oversight	All animal-related procedures were reviewed and approved by the Institutional Animal Care and Use Committee, Chungnam National University School of Medicine, Daejeon, Korea (CNU-00944)

Note that full information on the approval of the study protocol must also be provided in the manuscript.

Flow Cytometry

Plots

Confirm that:

☒ The axis labels state the marker and fluorochrome used (e.g. CD4-FITC).

☒ The axis scales are clearly visible. Include numbers along axes only for bottom left plot of group (a 'group' is an analysis of identical markers).

☒ All plots are contour plots with outliers or pseudocolor plots.

☒ A numerical value for number of cells or percentage (with statistics) is provided.

Methodology

Sample preparation	Primary BMDMs were isolated from the femurs of Mfn2 WT and Mfn2 CKO mice and cultured for 3 days in 25 ng ml−1 macrophage colony-stimulating factor (R&D Systems, Minneapolis, MN, USA). The culture medium used was Dulbecco's modified Eagle's medium (DMEM) supplemented with 10% heat-inactivated fetal bovine serum, 1 mM sodium pyruvate, 50 U ml−1 penicillin and 50 µg ml−1 streptomycin, which were purchased from Lonza (Walkersville, MD, USA).
Instrument	We used FACS Canto II flow cytometer (Becton Dickinson, San Jose, CA, USA)
Software	Flow cytometry data were collected and analyzed using FlowJo software (Tree Star, Ashland, OR, USA).
Cell population abundance	The abundance of the relevant cell populations was 10,000 cells per case.
Gating strategy	In our study, all FACS analysis was performed in total bone marrow-derived macrophages (BMDMs). The purity of the BMDM samples was initially determined using by anti-F4/80 and anti-CD11b for BMDMs, and always more than 95%. After the exclusion of debris, total BMDMs were further gated for F4/80-positive macrophages.

☒ Tick this box to confirm that a figure exemplifying the gating strategy is provided in the Supplementary Information.
